# Supplementary material for: Singlet fission dynamics modulated by molecular configuration in covalently linked pyrene dimers, Anti- and Syn-1,2-di(pyrenyl)benzene
Source: Commun Chem. 2023 Jan 17;6:16. doi: 10.1038/s42004-023-00816-6 (PMC9845327; doi:10.1038/s42004-023-00816-6)
Supplement: Supplementary file 2 — Supplementary Information [file 42004_2023_816_MOESM2_ESM.pdf]

Supplementary Information

Singlet Fission Dynamics Modulated by Molecular Configuration in Covalently Linked Pyrene Dimers, *Anti*- and *Syn*-1,2-di(pyrenyl)benzene

Jungkweon Choi,[a,b] *Siin Kim,*[a,b] *Mina Ahn,*[c] *Jungmin Kim,* [a,b] *Dae Won Cho,*[a,b] *Doyeong Kim,* [a,b] *Seunghwan Eom,*[a,b] *Donghwan Im,*[a,b] *Yujeong Kim,*[d] *Sun Hee Kim,*[d] *Kyung-Ryang Wee,** [c] *Hyotcherl Ihee** [a,b]

[a] Center for Advanced Reaction Dynamics, Institute for Basic Science, Daejeon 34141, Republic of Korea

[b] Department of Chemistry and KI for the BioCentury, Korea Advanced Institute of Science and Technology (KAIST), Daejeon 34141, Republic of Korea

[c] Department of Chemistry and Institute of Natural Science, Daegu University, Gyeongsan 38453, Republic of Korea

[d] Western Seoul Center, Korea Basic Science Institute (KBSI), Seoul 03759, Republic of Korea

* Corresponding authors: Kyung-Ryang Wee and Hyotcherl Ihee

E-mail: krwee@daegu.ac.kr, hyotcherl.ihee@kaist.ac.kr

Supplementary Methods

General: Based on standard Schlenk techniques, all of synthesis experimental procedures were performed under a dry argon condition. Reagents and solvents were purchased from commercial sources and used as received without further purification, unless otherwise stated. Deuterated solvent for NMR experiments were obtained from Merck or Cambridge Isotope Lab. Inc. All synthesized compounds were characterized by ^{1}H-NMR or ^{13}C-NMR, and elemental analysis. The ^{1}H and ^{13}C spectra were recorded on a Bruker500 spectrometer operating at 500 and 125 MHz, respectively, and all proton and carbon chemical shifts were measured relative to internal residual chloroform (99.5% $CDCl_3$) from the lock solvent. The GC-MS analysis was performed using a highly sensitive Gas Chromatograph /Mass Selective Detector spectrometer (Agilent, 7890B-5977B GC/MSD). The elemental analyses (C, H) were performed using Thermo Fisher Scientific Flash 2000 series analyzer. The crystal structure was determined by a single-crystal X-ray diffractometer at the Western Seoul Center of Korea Basic Science Institute.

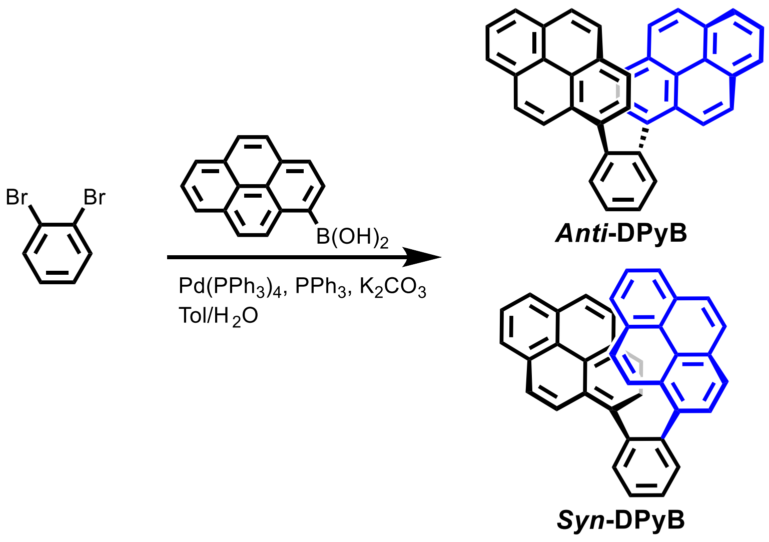

Supplementary Figure S1. Synthesis route to *anti*- and *syn*-1,2-di(pyrenyl)benzene (*Anti*-DPyB and *Syn*-DPyB).

Synthesis of *Anti*-DPyB and *Syn*-DPyB: A mixture of 1,2-dibromobenzene (1.0 g, 4.24 mmol), pyrene-1-boronic acid (3.13 g, 12.7 mmol), Pd(PPh3)4 (0.24 g, 5 mol%), K_2CO_3 (5.26 g, 38.1 mmol) in Toluene/H_2O (v/v = 50 mL/10 mL) was refluxed under argon at 110 °C for overnight. After cooling to room temperature, deionized water (50 ml) was poured and the organic layer was separated using a separating funnel. The water layer was washed using methylene chloride (×3) for extracted remaining organic residue. After combining all of the organic solvents, the organic layer was dried over anhydrous $MgSO_4$ and then filtered off. The solvent was removed under reduced pressure, and the residue was purified by silica gel column chromatography using

CH_2Cl_2/*n*-hexane (v/v = 1:6) as the eluent and obtained a mixture powder. *Anti*-DPyB was purified by recrystallization in CH_2Cl_2/*n*-hexane (v/v = 1:2) to give a pale yellow powder. Yield: 0.38 g, 19 %. ^{1}H NMR (500 MHz, $CDCl_3$, ppm) δ 8.30 (d, 1H), 8.22 (d, 1H), 8.18-8.12 (m, 3H), 8.03-8.00 (m, 2H), 7.95 (d, 2H), 7.90-7.83 (m, 3H), 7.79-7.69 (m, 5H), 7.57 (d, 1H), 7.47 (d, 1H). ^{13}C NMR (125 MHz, CDCl3, ppm) δ 141.0, 136.8, 136.6, 132.5, 132.4, 131.3, 131.1, 130.9, 130.7, 130.0, 130.0, 129.3, 128.8, 128.7, 127.8, 127.4, 127.3, 127.3, 127.2, 127.2, 127.0, 127.0, 126.8, 125.8, 125.6, 125.6, 125.5, 125.0, 124.8, 124.7, 124.6, 124.6, 124.5, 124.0, 123.7. DIP-MS Calcd for [C38H22]: 478.17 m/z, Found: 478.3 m/z. Elem. Anal. Found (Calcd) for C38H22: C, 95.36 (95.37); H, 4.64 (4.63). The recrystallized filtrate was evaporated under reduced pressure further purified by silica gel column chromatography using n-hexane as the eluent to obtain *Syn*-DPyB of light yellow powder. Yield: 0.22 g, 11 %. ^{1}H NMR (500 MHz, $CDCl_3$, ppm) δ 8.40-8.38 (d, 2H), 8.27-8.22 (m, 4H), 8.20-8.15 (m, 5H), 8.09-8.05 (m, 3H), 7.97 (t, 1H), 7.92 (d, 1H), 7.76-7.65 (m, 5H), 6.71 (t, 1H), 6.25 (d, 1H). ^{13}C NMR (125 MHz, $CDCl_3$, ppm) δ 141.9, 138.8, 135.3, 134.3, 134.3, 133.4, 133.3, 132.8, 131.4, 131.1, 130.7, 130.6, 130.5, 128.4, 128.0, 127.9, 127.7, 127.6, 127.3, 126.9, 126.8, 126.7, 126.6, 126.2, 125.5, 125.4, 125.3, 125.0, 124.8, 124.4, 123.4, 121.2, 120.3, 120.1, 119.8. DIP-MS Calcd for [C38H22]: 478.17 m/z, Found: 476.2 m/z. Elem. Anal. Found (Calcd) for C38H22: C, 95.34 (95.37); H, 4.66 (4.63).

Preparation of single crystal *Anti*-DPyB: For the crystallization of *Anti*-DPyB, vacuum dried pure sample was taken in a vial and dissolved in 1:2 dichloromethane/*n*-hexane mixed solvent. Slow evaporation of the solvent at room temperature for one month yielded the transparent square crystal, then was picked up from the vial and performed single crystal XRD study.

X-ray crystal structure analysis: The data were collected at 223(2) K using a Bruker D8 Venture equipped with IμS micro-focus sealed tube Mo Kα (λ= 0.71073 Å) and a PHOTON II 14 detector in Western Seoul Center of Korea Basic Science Institute. Preliminary unit cell constants were determined using a set of 45 narrow-frame (0.3° in ω) scans. The double pass method of scanning was used to exclude noise. Collected frames were integrated using an orientation matrix determined from narrow-frame scans. The SMART software package was used for data collection, and SAINT was used for frame integration.[1] Final cell constants were determined by global refinement of xyz centroids of reflections harvested from the entire data set. Structure solution and refinement were carried out using the SHELXTL-PLUS software package.[2]

Spectroscopic measurements: The steady-state UV-visible absorption and emission spectra of *Anti*-DPyB and *Syn*-DPyB were measured using a Shimadzu UV-2600 and FL spectrometer LS 55 (Perkin-Elmer), respectively. The femtosecond time-resolved absorption spectra were collected with a pump–probe transient absorption spectroscopy system. The output pulses at a wavelength of 800 nm from a Ti:sapphire amplified laser (Coherent Legend Elite) were split into the pump and probe beams. On the pump arm, the laser pulses of 800 nm were converted into the pump pulses of the wavelength of 345 nm using an optical parametric amplifier (Spectra Physics, OPAS prime). A white-light continuum pulse, which was generated by focusing the residual of the

fundamental light onto a 1 mm path length quartz cell containing water, was used as a probe beam. The white light was directed to the sample cell with an optical path of 2.0 mm and detected with a CCD detector installed in the absorption spectroscopy system after the controlled optical delay. The pump pulse was chopped by a mechanical chopper synchronized to one-half of the laser repetition rate, resulting in a pair of spectra with and without the pump, from which the absorption change induced by the pump pulse was estimated.

Fluorescence excitation spectra: We also checked the possibility that Py molecules are present as impurities in *Anti*-DPyB and *Syn*-DPyB solutions. Py molecule shows a strong fluorescence in solutions. Therefore, if Py molecules are present as impurities in *Anti*-DPyB and *Syn*-DPyB solutions, they may contaminate the fluorescence spectra from the *Anti*-DPyB and *Syn*-DPyB samples. To check this possibility, we measured the fluorescence excitation spectra of *Anti*-DPyB and *Syn*-DPyB in acetonitrile at two emission peak positions (380 and 480 nm). As can be seen in Supplementary Figure S8, the fluorescence excitation spectra from *Anti*-DPyB and *Syn*-DPyB are significantly different from the absorption spectrum of Py molecule. This result indicates that Py molecules do not exist as impurities in *Anti*-DPyB and *Syn*-DPyB solutions. Although the fluorescence excitation spectra at the two peak emission positions for *Anti*-DPyB are similar (Supplementary Figure S8A), their maximum peak positions are different; 329 nm for the 380-nm fluorescence excitation spectra and 344 nm for the 480-nm one. Accordingly, the two fluorescence excitation spectra have different ratios of the intensity at 329 nm to that at 344 nm, which suggests that two emission bands of 380 and 480 nm originate from two different emissive states. In addition, the two fluorescence excitation spectra are similar to the absorption spectrum of *Anti*-DPyB. In contrast to *Anti*-DPyB, the fluorescence excitation spectra measured at the two emission wavelengths (380 and 480 nm) of *Syn*-DPyB originating from the two emissive states (monomeric S_1 and excimer states) are significantly different from each other (Supplementary Figure S8B). The fluorescence excitation spectrum for the 380 nm emission is similar to the absorption spectrum of *Anti*-DPyB, which has a monomeric character, whereas the fluorescence excitation spectrum for the 480 nm emission is similar to the absorption spectrum of *Syn*-DPyB, respectively, indicating that two emission bands of 380 and 480 nm originate from two different emissive states. This result further supports our conclusion based on the emission spectra that the dual emissions (~380 and 480 nm) from *Anti*-DPyB and *Syn*-DPyB come from two emissive states, Py monomer moieties and the excimer.

Triplet quantum yield (Φ_T) of *Anti*-DPyB: We estimated the triplet quantum yield (Φ_T) of *Anti*-DPyB in *n*-hexane and acetonitrile using nanosecond TA spectroscopy. Φ_T was calculated using the following supplementary equation (S1),

$$\Phi_{\mathrm{T}} = \frac{\Delta A_{\mathrm{Sample}}}{\Delta A_{\mathrm{Ref}}} \frac{\varepsilon_{\mathrm{T\_Ref}}}{\varepsilon_{\mathrm{T\_Sample(Py)}}} \frac{Abs_{\mathrm{Ref}}}{Abs_{\mathrm{Sample}}} \cdot \Phi_{\mathrm{T\_Ref}} \qquad \text{(S1)}$$

where ΔA_{Sample} and ΔA_{Ref} are the delta absorbances of the sample and the reference measured by nanosecond TA experiment, respectively. Φ_T and Φ_{T_Ref} represent the triplet quantum yields of the sample and the reference sample, respectively. $\varepsilon_{T\_Sample(Py)}$ and ε_{T_Ref} are the triplet extinction coefficients of pyrene (Py) and the reference sample, respectively. Abs_{Sample} and Abs_{Ref} are the absorbances of the sample and the reference sample at 355 nm, respectively. The triplet quantum yield ($\Phi_{T\_Ref} = 1$) of benzophenone was used for Φ_{T_Ref}. It is known that the ε_T value of Py in benzene is 20900 M^{-1} cm^{-1} at 420 nm and the ε_T value of benzophenone in benzene is 7630 at 532.5 nm.[3] Since it is generally known that the effect of solvents on the triple extinction coefficient of a solute molecule is small, we used the ε_T values of Py and benzophenone measured in benzene to determine the Φ_T values of *Anti*-DPyB in *n*-hexane and acetonitrile. From the nanosecond TA experiment, the Φ_T values of *Anti*-DPyB in *n*-hexane and acetonitrile are determined to be 44.1 and 17.5%, respectively.

Time-resolved EPR spectroscopy: The TR-EPR measurements were carried out at Korea Basic Science Institute (KBSI) in Seoul, Korea. *Anti*-DPyB and *Syn*-DPyB were dissolved in toluene, and the sample concentrations were adjusted to 2 mM. The sample solutions were degassed by purging with N_2 gas for 1.5 h and transferred to EPR tubes via cannula using Schlenk techniques under an N_2 atmosphere. The transferred solutions were immediately frozen in liquid N_2. The photoexcitations were performed by the third harmonics (355 nm) of a nanosecond Q-switched Nd:YAG laser (Continuum, Surelite-I). The laser power used in this work was 6 mJ at the 10 Hz repetition rate. Time-resolved EPR data were obtained on a Bruker Elexsys E580 spectrometer, and the cryogenic temperature was achieved with an Oxford CF-935 cryostat and Oxford ITC temperature controller. X-band (9.728 GHz) transient EPR data were acquired using a Bruker 4118X-MD5 dielectric ring resonator and Q-band (34 GHz) time-resolved pulsed EPR were collected using an EN5107D2 resonator.

X-band CW (continuous wave) transient EPR experiments were performed by direct detection at a temperature of 80 K with a microwave power of 15 mW. The laser was triggered by an external digital pulse delay generator, and the delay times were adjusted to 160-200 ns. The background signal from the laser was removed by 2D baseline correction determined based on the off-resonance transients. 34 GHz time-resolved ESE (electron spin echo) detected spectra were carried out using the pulse sequence, laser-T_{delay}-π/2-τ-π-τ-echo, with microwave pulse lengths of 16-32 ns and an inter-pulse time of τ = 200 ns. The pulsed EPR measurements were conducted at 80 K. All experimental spectra were simulated using EasySpin.[4]

Supplementary Figure S20 shows EPR spectra of *Anti*-DPyB and *Syn*-DPyB at 128 and 200 ns after photoirradiation. The EPR signals for *Anti*-DPyB and *Syn*-DpyB show the narrow peak splitting of 34 and 19 mT around 340 mT (g = 2.002), respectively. In addition to the narrow peak splitting, *Anti*-DPyB and *Syn*-DPyB exhibit a large peak splitting of 150 and 115 mT, respectively. The EPR signal for *Anti*-DPyB is well reproduced by the simulated curve for its triplet using EasySpin with zero-field splitting (ZFS) parameters of *D* = -2399 MHz and *E* = 480 MHz. Similarly, the EPR signal for *Syn*-DPyB is well reproduced by the simulated curve for its triplet using EasySpin with ZFS parameters of *D*

= -1890 MHz and E = 450 MHz. These consistencies suggest that the EPR signals measured from *Anti*-DPyB and *Syn*-DPyB arise from triplet (S = 1) species. To further confirm the origins of TR-EPR signals, the nutation experiment for Q-band (34 GHz) TR-EPR signal of *Anti*-DPyB was measured using the pulse sequence, laser-T_{delay}-π/2-τ-π-τ-echo, with microwave pulse lengths of 16 - 32 ns and an inter-pulse time of τ = 200 ns. As shown in Supplementary Figure S21A, Q-band (34 GHz) TR-EPR spectrum of *Anti*-DPyB shows a microwave emission (*E*)/absorption (*A*) polarized pattern similar to X-band EPR spectrum. It is known that in the extremely weak limit of the microwave irradiation field ($B_1 = w_1/g$), the nutation frequency w_n of spin magnetization is simply given by $w_n = w_1[S(S + 1) - m_s(m_s - 1)]^{1/2}$ for a transition $|S, m_s\rangle \leftrightarrow |S, m_s - 1\rangle$. In this experiment, w_1 is 8.5 MHz. The w_ns for EPR peaks of 1131.5, 1195.3,1229.7, and 1293.0 mT are determined to be all 11.74 MHz. The observed nutation frequency ratios w_n/w_1 are ~1.4, consistent with the theoretical value corresponding to the $T_0 \rightarrow T_{\pm1}$ transition. This result supports that the EPR signal measured from *Anti*-DPyB is attributed to triplet species. Although the nutation measurement on the EPR signal of *Syn*-DPyB was not performed, we speculate that the X-band EPR signal measured from *Syn*-DPyB arises from triplet species as well. The EPR signals at a few hundred nanoseconds do not show evidence for (T_1T_1). The absence of EPR signals of (T_1T_1) for *Anti*-DPyB and *Syn*-DPyB at a few hundred nanoseconds is probably due to the shorter lifetimes of (T_1T_1)s than the temporal resolution (~120 ns) of our TR-EPR system. The femtosecond TA measurements show that the time profile for transient absorption bands of *Anti*-DPyB around 440 nm, which well reflects the relaxation kinetics of (T_1T_1), shows slow but distinct rising features (Supplementary Figure S19A), suggesting that the lifetime of (T_1T_1) for *Anti*-DPyB is longer than 10 ns. Compared with *Anti*-DPyB, *Syn*-DPyB shows a relatively fast decay feature in the time profile for transient absorption bands of 450 nm (Supplementary Figure S19B). As shown in Table 2, the (T_1T_1)s of *Syn*-DPyB in *n*-hexane and acetonitrile relax to $2T_1$ and S_0 in parallel with time constants of 6.4 and 4.8 ns, respectively, indicating that the lifetime of (T_1T_1) for *Syn*-DPyB should be significantly shorter than the temporal resolution (~120 ns) of our TR-EPR system. Meanwhile, we could not precisely determine the lifetime of (T_1T_1) for *Anti*-DPyB because of the limited range of investigated delay times in the femtosecond TA measurement. Overall, the EPR data lead us to conclude that the lifetime of (T_1T_1) for *Anti*-DPyB should be shorter than the temporal resolution (~120 ns) of our TR-EPR system.

Singular value decomposition (SVD) analysis: We applied the SVD analysis to our experimental data in the λ range of 400–700 nm. From the experimental TA spectra measured at various time delays, we can build an $n_\lambda \times n_t$ matrix **A**, where n_λ is the number of λ points in the TA spectrum at a given time-delay point (253 wavelength points for *Anti*-DPyB and *Syn*-DPyB) and n_t is the number of time-delay points (662 time delay points in the wavelength range from 400 nm to 700 nm for *Anti*-DPyB and *Syn*-DPyB). Then, the matrix **A** can be decomposed while satisfying the relationship of $\mathbf{A} = \mathbf{USV}^T$, where **U** is an $n_\lambda \times n_t$ matrix whose columns are called left singular

vectors (lSVs) (i.e. time-independent λ spectra) of **A**, **V** is an $n_t \times n_t$ matrix whose columns are called right singular vectors (rSVs) (i.e. amplitude changes of **U** as time evolves) of **A**, and **S** is an $n_t \times n_t$ diagonal matrix whose diagonal elements are called singular values of **A** and can possess only non-negative values. The matrices **U** and **V** have the properties of $\mathbf{U}^T\mathbf{U} = \mathbf{I}_{nt}$ and $\mathbf{V}^T\mathbf{V} = \mathbf{I}_{nt}$, respectively, where $\mathbf{I}_{nt}$ is the identity matrix. Since the diagonal elements (i.e. singular values) of **S**, which represent the weight of left singular vectors in **U**, are ordered so that $s_1 \geq s_2 \geq \cdots\cdots \geq s_n \geq 0$, (both left and right) singular vectors on more left are supposed to have larger contributions to the constructed experimental data. In this manner, we can extract the time-independent transient absorption components from the lSVs and the time evolution of their amplitudes from the rSVs. The former, when combined together, can give information on the TA spectra of distinct transient species, while the latter contains information on the population dynamics of the transient species.

The singular values and autocorrelations of the corresponding singular vectors suggest that the first n_p singular vectors are enough to represent our experimental data because the contribution of each singular vector (lSV or rSV) to the data is proportional to its corresponding singular value and the autocorrelation of **U** or **V** matrix can serve as a good measure of the signal-to-noise ratio of the singular vectors (in this study, four and three significant singular components for the data of *Anti*-DPyB and *Syn*-DPyB, respectively). In other words, the contribution from the $(n_p + 1)_{\text{th}}$ singular vectors and beyond becomes negligible. The SVD analysis results are shown in Figure S11 (*Anti*-DPyB in *n*-hexane and acetonitrile), Supplementary Figure S12 (*Syn*-DPyB in *n*-hexane and acetonitrile).

To extract kinetic information, as many rSVs as n_p multiplied by singular values were fit by a sum of multiple exponentials sharing common relaxation times as follows:

$$s_o V_{o,fit} = c_o + \sum_{i=1}^{m} A_{i,o} e^{-t/t_i} \quad \text{(S2)}$$

where s_o is *o*th singular value,$V_{o,\text{fit}}(t)$ are *o*th rSVs try to fit, *t* are time delays, c_n is constant for $V_{o,\text{fit}}(t)$ offset, *m* is the number of exponential functions, $A_{i,o}$ is amplitude for *i*th exponential of $V_{o,\text{fit}}(t)$, t_i is *i*th sharing relaxation times. The $V_{o,\text{fit}}(t)$ are optimized by minimizing discrepancy (quantified by the test function, TF), which is the sum of the every residual between $V_{o,\text{fit}}(t)$ and *o*th rSVs, $V_o(t)$, as following:

$$TF = \sum_{t}^{n_t} \sum_{o=1}^{n_p} \left| s_o V_o(t) - s_o V_{o,fit}(t) \right| \quad \text{(S3)}$$

To find an appropriate number of exponentials, we performed the fitting by changing the number of exponentials. In the case of *Anti*-DPyB, the slowest exponential relaxation time did not converge during fitting due to the limited range of time delays, so the slowest relaxation time was fixed to 10 ns. The first four rSVs were simultaneously fitted with a sum of exponential functions with shared relaxation times. A tetra-exponential functions with the shared time constants of 3.6 ± 0.3 ps, 231 ± 19 ps, 1.75 ± 0.12 ns, and >10 ns in *n*-hexane and 2.8 ± 0.1 ps, 24.3 ± 0.5 ps, 495.7 ± 6.5 ps, and >10 ns in acetonitrile gave satisfactory fits as shown in Supplementary Figures S13A and S131B, respectively. $V_{o,\text{fit}}(t)$ with less than four exponential functions could not provide a satisfactory fit to V_o for *Anti*-DPyB. $V_{o,\text{fit}}(t)$ with more than four exponential functions could fit

V_o, but some exponential time constants show no meaningful difference, indicating that they were overfitted. The obtained relaxation time are 3.6 ± 0.3 ps, 231 ± 19 ps, 1.75 ± 0.12 ns, and > 10 ns in *n*-hexane and 2.8 ± 0.1 ps, 24.3 ± 0.5 ps, 495.7 ± 6.5 ps, and > 10 ns in acetonitrile. In the case of *Syn*-DPyB, the first three rSVs were simultaneously fitted with a sum of exponential functions with shared relaxation times. A tri-exponential functions with the shared time constants of 2.3 ± 0.8 ps, 9.7 ± 0.5 ps, and 6.4 ± 0.2 ns in n-hexane; 2.8 ps, 8.0 ± 0.6 ps, and 4.8 ± 0.2 ns in acetonitrile gave satisfactory fits as shown in Supplementary Figures S13C and S13D, respectively. $V_{o,fit}(t)$ with less than three exponential functions could not provide a satisfactory fit to V_o for Anti-DPyB. $V_{o,fit}(t)$ with more than four exponential functions could fit V_o, but some exponential time constants show no meaningful difference, indicating that they were overfitted. As mentioned in the main text, the obtained relaxation time are 2.3 ± 0.8 ps, 9.7 ± 0.5 ps, and 6.4 ± 0.2 ns in n-hexane; 2.8 ps, 8.0 ± 0.6 ps, and 4.8 ± 0.2 ns in acetonitrile.

Kinetic analysis: Using the first a few singular vectors of significant singular values (that is, n_p principal singular vectors) obtained from the SVD analysis of the experimental data, we performed kinetic analysis. New matrices, **U**', **V**', and **S**', can be defined by removing non-significant components from **U**, **V**, and **S**, respectively. In other words, **U**' is an $n_\lambda \times n_p$ matrix containing the first n_p left singular vectors of **U**, **V**' is an $n_t \times n_p$ matrix containing the first n_p right singular vectors of **V**, and **S**' is an $n_p \times n_p$ diagonal matrix containing the first n_p singular values of **S**. Here we represent the time-dependent concentrations of transiently formed intermediate species, which can be calculated from a kinetic model, by a matrix **C**. Then, the matrix **C** can be related to **V**' by using a parameter matrix **P** that satisfies **V**' = **CP**, where **C** is an $n_t \times n_p$ matrix whose columns represent time-dependent concentrations of transiently formed intermediate species and **P** is an $n_p \times n_p$ matrix whose columns contain coefficients for the time-dependent concentrations so that the linear combination of concentrations of the n_p intermediates can form the n_p right singular vectors in **V**', respectively. Once **C** is specified by a kinetic model with a certain set of variable kinetic parameters such as rate coefficients, **P** and **C** can be optimized by minimizing the discrepancy between **V**' (from the experiment) and **CP** (from the kinetic theory).

Since **V**' = **CP**, the following relationships hold:

$$\mathbf{A}' = \mathbf{U}'\mathbf{S}'\mathbf{V}'^{\mathrm{T}} = \mathbf{U}'\mathbf{S}'(\mathbf{CP})^{\mathrm{T}} = \mathbf{U}'\mathbf{S}'\mathbf{P}^{\mathrm{T}}\mathbf{C}^{\mathrm{T}} = (\mathbf{U}'\mathbf{S}'\mathbf{P}^{\mathrm{T}})\mathbf{C}^{\mathrm{T}} \tag{S4}$$

where **A**' is an $n_\lambda \times n_t$ matrix that contains the theoretical TA spectrum $\Delta A(\lambda_i,t_j)$ at given λ and t values. Theoretical TA spectra calculated by using Supplementary Equation (S4) were compared with the experimental TA spectra, and the matrix **P** and **C** were optimized by minimizing the discrepancy (quantified by least-square, LS) between the theoretical and experimental TA spectra using the Minuit[1] package:

$$LS = \sum_{i=1}^{n_\lambda}\sum_{j=1}^{n_t}\left\{A_{\exp}(\lambda_i,t_j) - A_{the}(\lambda_i,t_j)\right\}^2 \tag{S5}$$

$\Delta A_{exp}(\lambda_i,t_j)$ and $\Delta A_{the}(\lambda_i,t_j)$ are the experimental and theoretical TA spectrum at a given point of (λ_i,t_j), respectively. From Supplementary Equation (S4), we can define a matrix **B** as $\mathbf{B} = \mathbf{U'S'P}^T$, that is, a linear combination of the n_p left singular vectors in **U**' weighted by their singular values in **S**' with their ratios determined by **P**. Then, the matrix **E**, an $n_t \times n_p$ matrix, contains the n_p time-independent TA spectra directly associated with the n_p intermediate species. Therefore, by optimizing the matrices P and C, we obtain both the time-dependent concentrations (see the optimized **C** for the kinetic model in Figures 4C, 4D, 5C and 5D) and the time-independent TA spectra of the intermediate species (see the optimized **P** for the kinetic model in Figures 5A, 5B, 6A and 6B).

For the kinetics analysis of the TA spectra for *Anti*-DPyB and *Syn*-DPyB, we considered various plausible kinetic models. For *Anti*-DPyB, considering five principal components from SVD analysis (Supplementary Figure S11) and four time constants obtained from the fitting of rSVs (Figure S13), we can set up the simplest kinetic model with five intermediates assigned to FC, S_1, excimer, (T_1T_1), and $2T_1$ and four time constants connecting them. In this kinetic model, the molecules in the (T_1T_1) state decay only to $2T_1$. In fact, they can also decay to S_0 as well, and such a case is also compatible with the SVD results. Therefore, one more time constant for the transition from (T_1T_1) to S_0 should be added. The resulting kinetic model is Kinetic Model (1) in Figure 4 of the main text. As the SVD results showed that the lifetime of (T_1T_1) is longer than 10 ns, we fixed two time constants for the process from (T_1T_1) to the ground state (τ_4) and the dissociation process of (T_1T_1) to free triplets (τ_5) as a sufficiently large number, 10 ns. Five SADS curves, population changes for five intermediates (FC, S_1, excimer, (T_1T_1), and $2T_1$), the experimental TA spectra, the best-fit spectra, and the residuals between them for *Anti*-DPyB in *n*-hexane and acetonitrile are shown in Supplementary Figures S22 and S23, respectively. The residuals are small, suggesting that the measured TA spectra for *Anti*-DPyB are well constructed as a linear combination of the five SADS curves.

While Kinetic Model (1) can explain the TA data well, it cannot explain the emission behavior, three time constants and emission quantum yields from emission experiments (Table 1). The fluorescence decay profiles showed two time constants assigned to the fluorescence lifetimes of the Py monomeric unit and excimer. By adding these two fluorescence decay times to Kinetic Model (1), we set up a new kinetic model (Kinetic Model (2) in Figure 4 of the main text). In this kinetic model, we also included the backreaction from the (T_1T_1) to the excimer for the following reason. As shown in the inset of Figure 2A, the rising time of 1.24 ns in the fluorescence decay profile for *Anti*-DPyB in *n*-hexane is approximately five times larger than the time constant (0.23 ns) corresponding to the $S_1 \rightarrow$ excimer transition determined from femtosecond TA experiments. This difference indicates that the observed excimer fluorescence is not the prompt emission but the delayed emission, suggesting the equilibrium between the excimer state and the (T_1T_1) state. In summary, Kinetic Model (2) has seven time constants and five intermediates. For the backreaction from (T_1T_1) to the excimer in *n*-hexane, the time constant of 1.24 ns was used. Unlike in *n*-hexane, no rising feature was observed in acetonitrile, implying that the process from (T_1T_1) state to excimer state is faster than the temporal resolution (~50 ps) of our time-resolved

fluorescence measurement system. Thus, we assumed that the backreaction process from (T_1T_1) to the excimer in acetonitrile occurs with a time constant of 50 ps. Figure 4 shows the five SADS curves and population changes for five intermediates (FC, S_1, excimer, (T_1T_1), and $2T_1$), and Supplementary Figure S24 shows the experimental TA spectra, the best-fit spectra, and the residuals between them for *Anti*-DPyB in *n*-hexane and acetonitrile obtained from the kinetic analysis using Kinetic Model (2). The residuals between the experimental and the best-fit spectra are small, suggesting that the measured TA spectra for *Anti*-DPyB are well constructed as a linear combination of the five SADS curves according to the employed kinetic model (Kinetic Model (2)). As the fit qualities of both Kinetic Model (1) and Kinetic Model (2) are comparable, fit qualities alone cannot tell which kinetic model is better. As discussed above and in the main text, however, Kinetic Model (2) is preferred because it is more consistent with the emission data.

For *Syn*-DPyB, considering the three exponential time constants obtained from the exponentials fitting of rSVs (Supplementary Figure S13), the two time constants and emission quantum yields from emission experiments (Table 1), and four principal components from SVD analysis results (Supplementary Figure S12), we can preferentially set up a kinetic model with five time constants and four intermediates. In addition, the TR-EPR signal for *Syn*-DPyB indicates that the (T_1T_1) of *Syn*-DPyB also dissociates to free triplets. Based on this result, the (T_1T_1) $\rightarrow$ $2T_1$ transition was included to the kinetic model. We also included the backreaction from (T_1T_1) to the excimer as in *Anti*-DPyB. Consequently, like *Anti*-DPyB, we used the kinetic model with five intermediates assigned to FC, S_1, excimer, (T_1T_1), and $2T_1$ (see Figure 4). In the case of *Syn*-DPyB, it is noteworthy that the τ_2 time constant corresponding to the S_1 → excimer transition was not resolved in *Syn*-DpyB (Table 2). As discussed in the main text, the excimer of *Syn*-DPyB with a pre-stacked structure is likely to form fast within a subpicosecond (≤ 200 fs) or with a time constant comparable to IVR (~3 ps). In this regard, we considered two kinetic models (Kinetic Models (3) and (4) in Figure 4 of the main text). In the former kinetic model, the S_1 → excimer transition occurs in subpicosecond (≤ 200 fs), and in the other kinetic model, the S_1 → excimer transition occurs with a time constant comparable to IVR (~3 ps). Five SADS curves, population changes for five intermediates (FC, S1, excimer, (T_1T_1), $2T_1$), and residuals from the kinetic analysis using Kinetic Model (3) are shown in Supplementary Figure S25. Figure 6 shows the five SADS curves and population changes for five intermediates (FC, S1, excimer, (T_1T_1), and $2T_1$) for *Syn*-DPyB obtained from the kinetic analysis for Kinetic Model (4) in *n*-hexane and acetonitrile. Supplementary Figure S26 shows the experimental TA spectra, the simulated spectra by a linear combination of the four SADS curves according to Kinetic Model (4), and residuals for *Syn*-DPyB in *n*-hexane and acetonitrile. As shown in Supplementary Figures S25 and S26, both Kinetic Models (3) and (4) show small residuals, suggesting that the measured TA spectra for *Syn*-DPyB are well constructed as linear combinations of the four SADS curves according to both kinetic models. Since the fit qualities of both kinetic models are comparable, fit qualities alone cannot tell which kinetic model is better. Nevertheless, the SADS curves from the two kinetic models are different and provide clues on which kinetic model is better. Whereas the SADS for the S_1 state from Kinetic Model (4) is positive (Figure 6), that from Kinetic Model (3) is strongly negative

(Supplementary Figure S21). Since a negative SADS is not possible for ESA (excited state absorption) from the S_1 state, Kinetic Model (3) can be ruled out. In other words, the kinetic analysis suggests that the $S_1 \rightarrow$ excimer transition in *Syn*-DPyB occurs with a time constant comparable to IVR (~3 ps).

Consideration for direct SF mechanisms from the S_1 state: It was also reported that the intermolecular and intramolecular SF dynamics could rapidly occur with a direct process from S_1 state to the free state due to strong coupling between the S_1 state and the free triplet state.[5, 6, 7] For example, Dover et al. suggested that the SF channel is dominated by a direct mechanism from the S_1 state and the formation of the excimer state inhibits the efficient SF dynamics.[7] Thus, we also checked the possibility that our data from *Anti*-DPyB can be explained with the same direct mechanism. Assuming that the assignment of the five intermediates to FC, S_1, excimer, (T_1T_1), and $2T_1$ is valid, we can consider a kinetic model that the direct SF process from the S_1 state to the free triplet state occurs with a time constant of τ_2. In this kinetic model (Kinetic Model depicted in Supplementary Figure S27), the second time constant (τ_2) observed from *Anti*-DPyB can be assigned to the SF dynamics from the S_1 state to free triplet state while other time constants of τ_1, τ_3, and τ_4 are assigned to the IVR, the $S_1 \rightarrow$ excimer transition, and the excimer $\rightarrow (T_1T_1)$ transition, respectively. The $(T_1T_1) \rightarrow 2T_1$ transition (τ_5) is excluded to fully reflect the nature of the direct SF process to form the free triplets directly from the S_1 state. We analyzed the TA spectra of *Anti*-DPyB in acetonitrile with this kinetic model (Kinetic Model depicted in Supplementary Figure S27). Supplementary Figure S28 shows the SADS curves, population changes for five intermediates (FC, S_1, excimer, (T_1T_1), and $2T_1$), and residuals for *Anti*-DPyB in acetonitrile according to Kinetic Model depicted in Supplementary Figure S27. The residuals between the experimental TA spectra and the best-fit spectra are not negligible (Supplementary Figure S28), meaning that unlike Kinetic Model (2), Kinetic Model (S1) involving a direct SF process does not reproduce well the measured TA spectra for *Anti*-DPyB in acetonitrile. This result indicates that the direct SF mechanism occurring with the τ_2 time constant does not reproduce well the measured TA spectra for *Anti*-DPyB in acetonitrile. The fit quality could be improved when in this kinetic model (Kinetic Model (S1)), the direct SF process from the S_1 state to the free triplet state is forced to occur with a low reaction yield (50%). But, the SADS for (T_1T_1) is strongly negative, which is not possible for ESA from (T_1T_1) (data not shown). Thus, this kinetic model can be ruled out. As another possibility, we also considered a kinetic model that the direct SF process and IVR in FC state simultaneously occur with a time constant of τ_1. In this kinetic model, the first time constant (τ_1) observed from *Anti*-DPyB can be assigned to the direct SF dynamics from the FC state to the free triplet state and IVR while other time constants ($\tau_2 - \tau_4$) are assigned as in the reaction mechanism we propose (Kinetic Model (2)). This direct SF mechanism from the FC state to the free triplet state occurring with the τ_1 time constant does not reproduce well the measured TA spectra for *Anti*-DPyB in acetonitrile (data not shown). These results indicate that the τ_1 and τ_2 time constants observed from *Anti*-DPyB cannot be attributed to the time constant for the direct SF process. If the SF dynamics in *Anti*-DPyB occurred with a direct mechanism, those CLDs would

be likely to show efficient SF dynamics due to the fast SF process from the S_1 state to the free triplet state. However, as explained in the main text, the SF dynamics of anti-DPyB shows the low triplet quantum yield in *n*-hexane and acetonitrile (44.1 in *n*-hexane and 17.5% in acetonitrile) and *Syn*-DPyB does not exhibit any absorption band in both *n*-hexane and acetonitrile, suggesting that the SF dynamics in *Anti*-DPyB and *Syn*-DPyB are not efficient. These results suggest that the SF dynamics of *Anti*-DPyB and *Syn*-DPyB cannot be explained by a direct SF mechanism.

The lack of the direct SF process in our data is also evident in the time profiles for transient absorption bands of *Anti*-DPyB around 440 nm, which corresponds to the T-T absorption band showing slow rising features (Figure S19A). Fitting the time profiles to an exponential function yields the rising times of > 10 ns and 461 ps in *n*-hexane and acetonitrile, respectively. These rising times indicate that in *Anti*-DPyB, the SF process to form free triplets occurs too slowly to be assigned to the direct SF process. The rising times (> 1 ns) are significantly slower than two time constants (τ_1 and τ_2) assigned τ_1 and τ_2 to the intramolecular vibrational relaxation (IVR) (τ_1 = ~3 ps) from the initially populated local excited state (FC state) and the $S_1 \rightarrow$ excimer transition (231 ps in *n*-hexane and 24.3 ps in acetonitrile), respectively. Consequently, our results are more consistent with the scenario that the SF dynamics in *Anti*-DPyB and *Syn*-DPyB proceed through an excimer-mediated process rather than a direct SF mechanism caused by strong coupling between the S_1 state and free triplet state. This result may indicate that the coupling between the S_1 state and free triplet state in CLDs such as *Anti*-DPyB and *Syn*-DPyB is weaker than in the molecules that showed such direct SF processes.

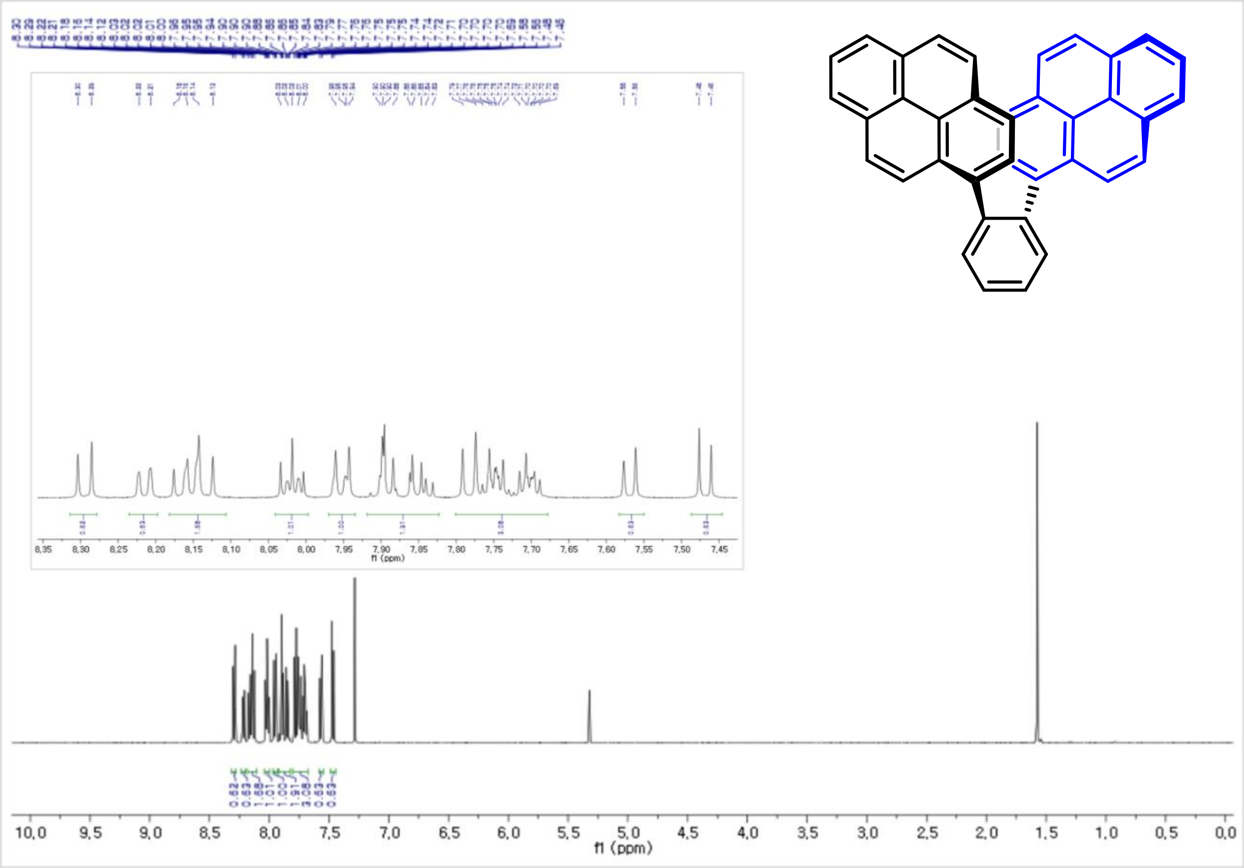

Supplementary Figure S2. ^{1}H-NMR spectrum of *Anti*-DPyB in $CDCl_3$.

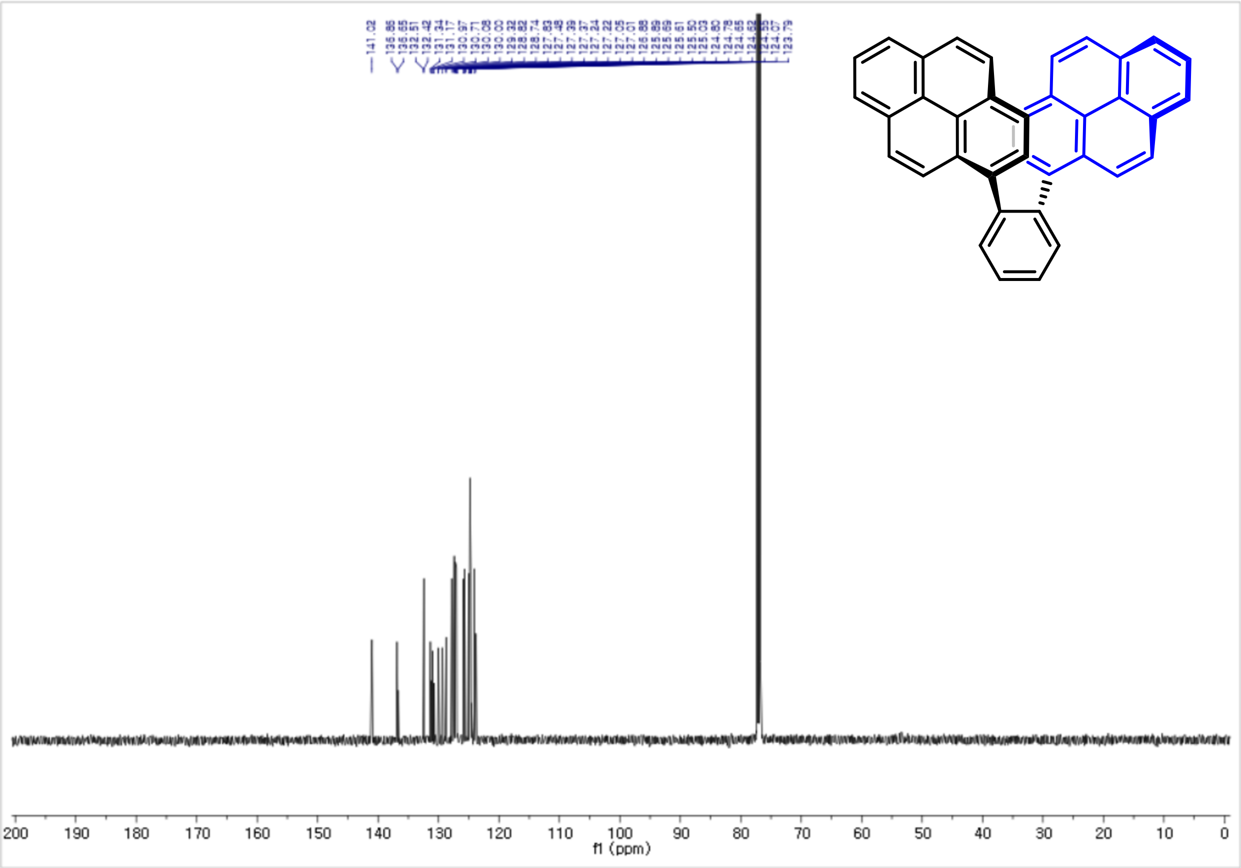

Supplementary Figure S3. ^{13}C-NMR spectrum of *Anti*-DPyB in $CDCl_3$.

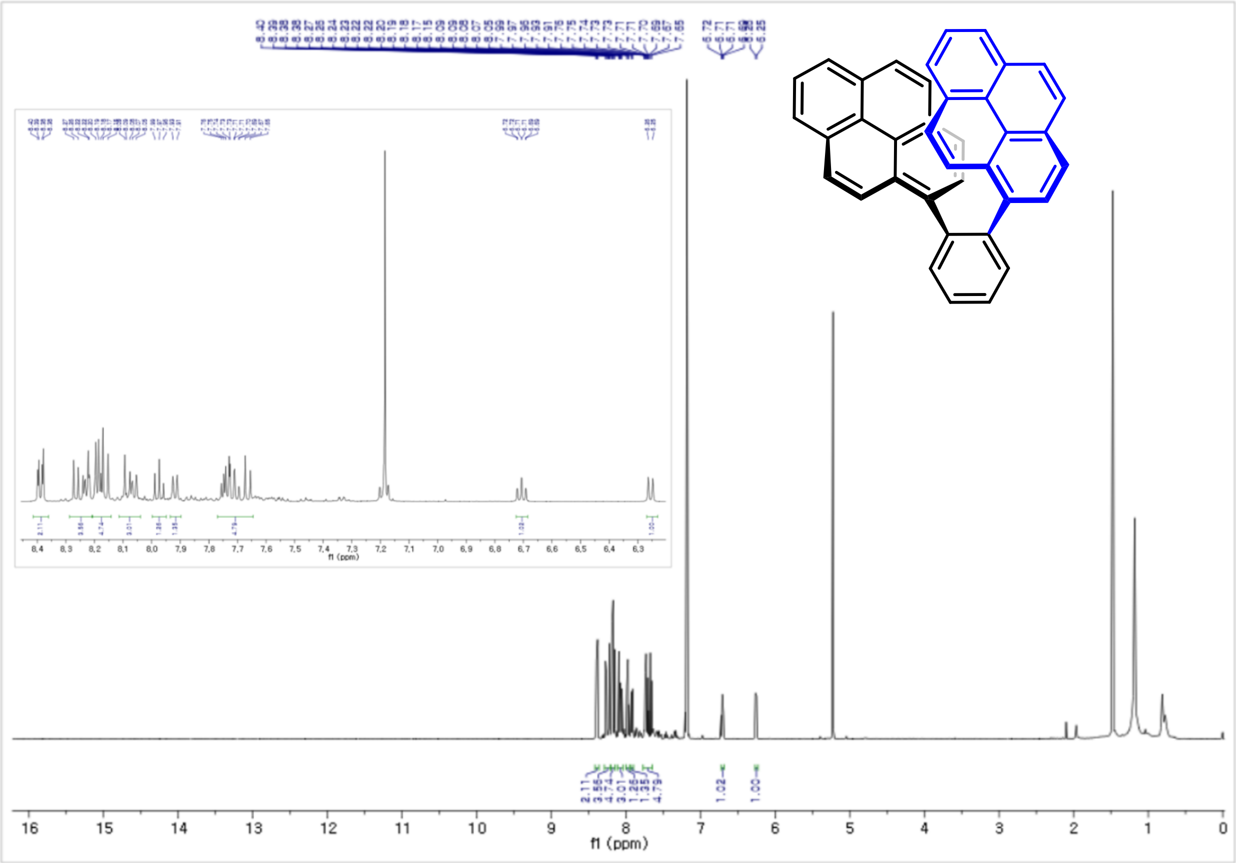

Supplementary Figure S4. ^{1}H-NMR spectrum of *Syn*-DPyB in $CDCl_3$.

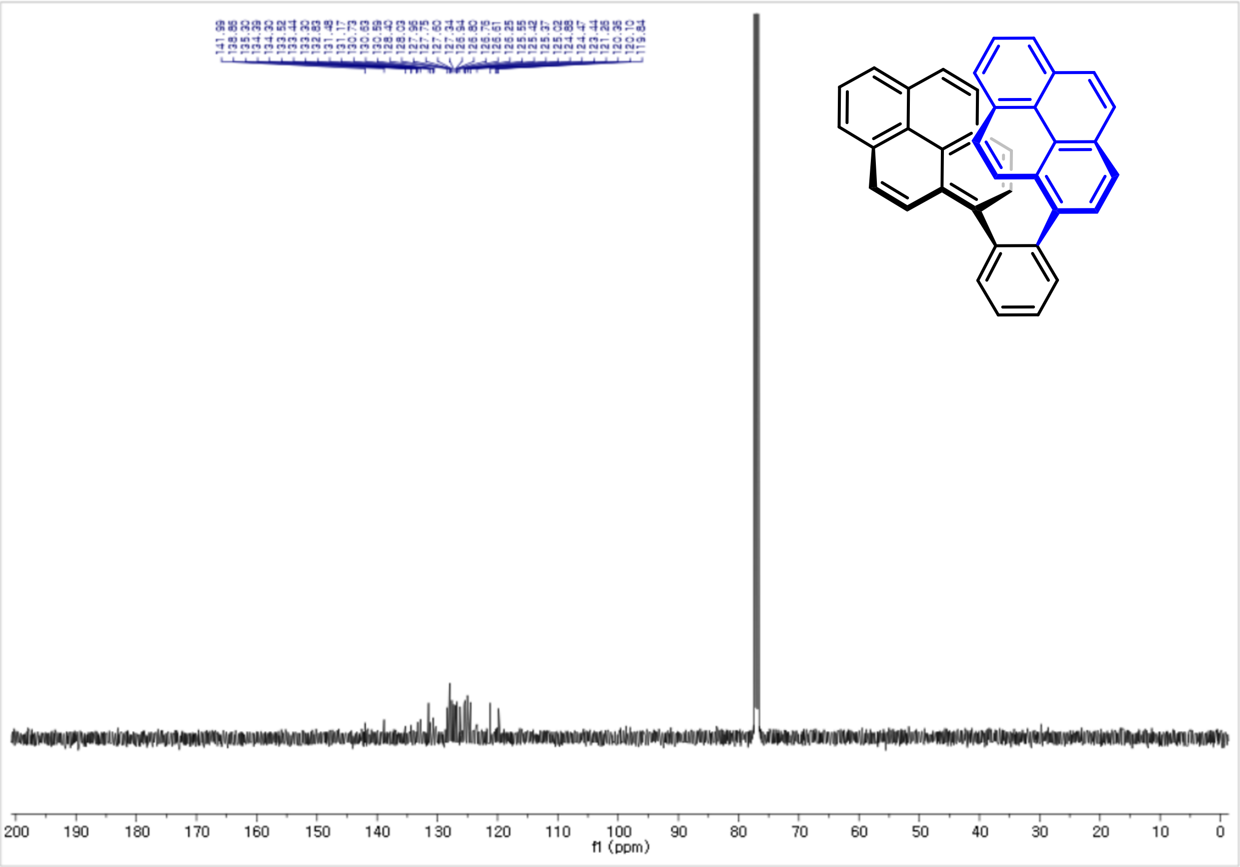

Supplementary Figure S5. ^{13}C-NMR spectrum of *Syn*-DPyB in $CDCl_3$.

A)

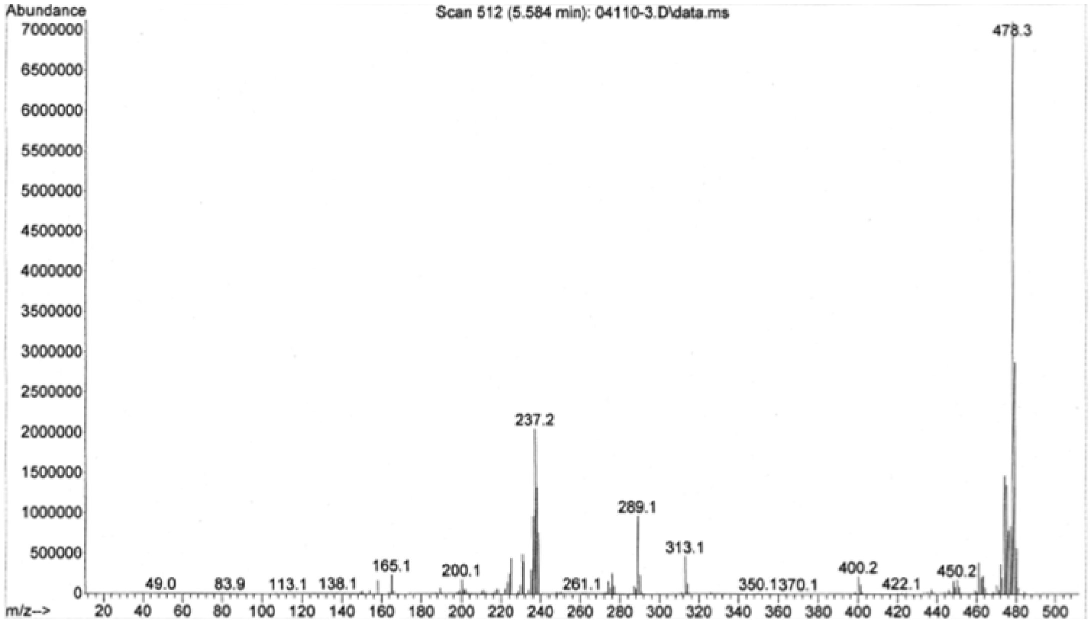

B)

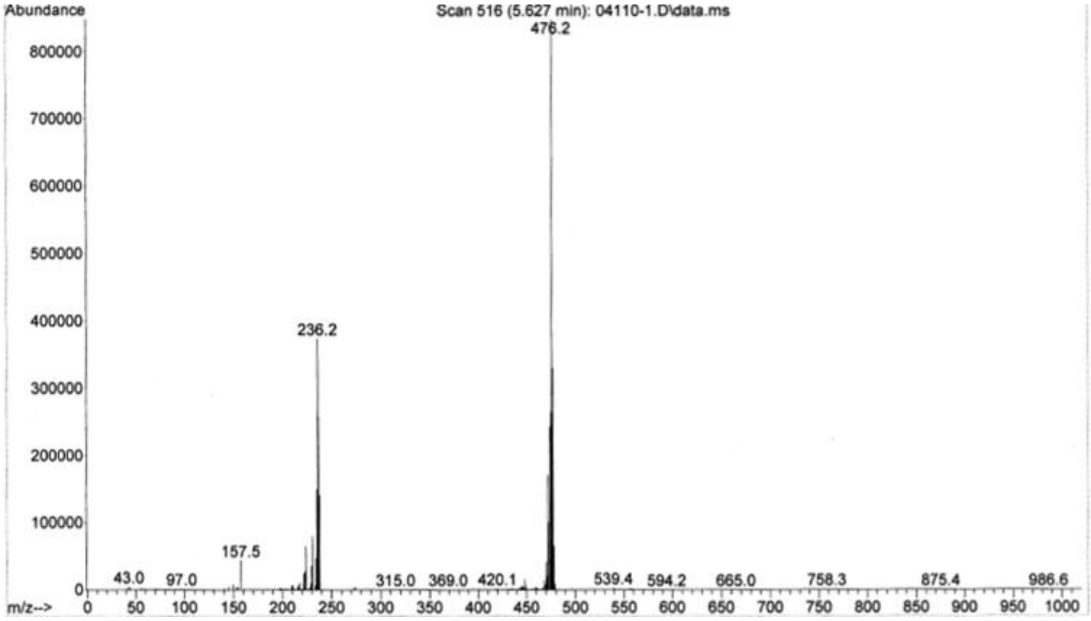

Supplementary Figure S6. GC-MS data of (A) *Anti*-DPyB and (B) *Syn*-DPyB.

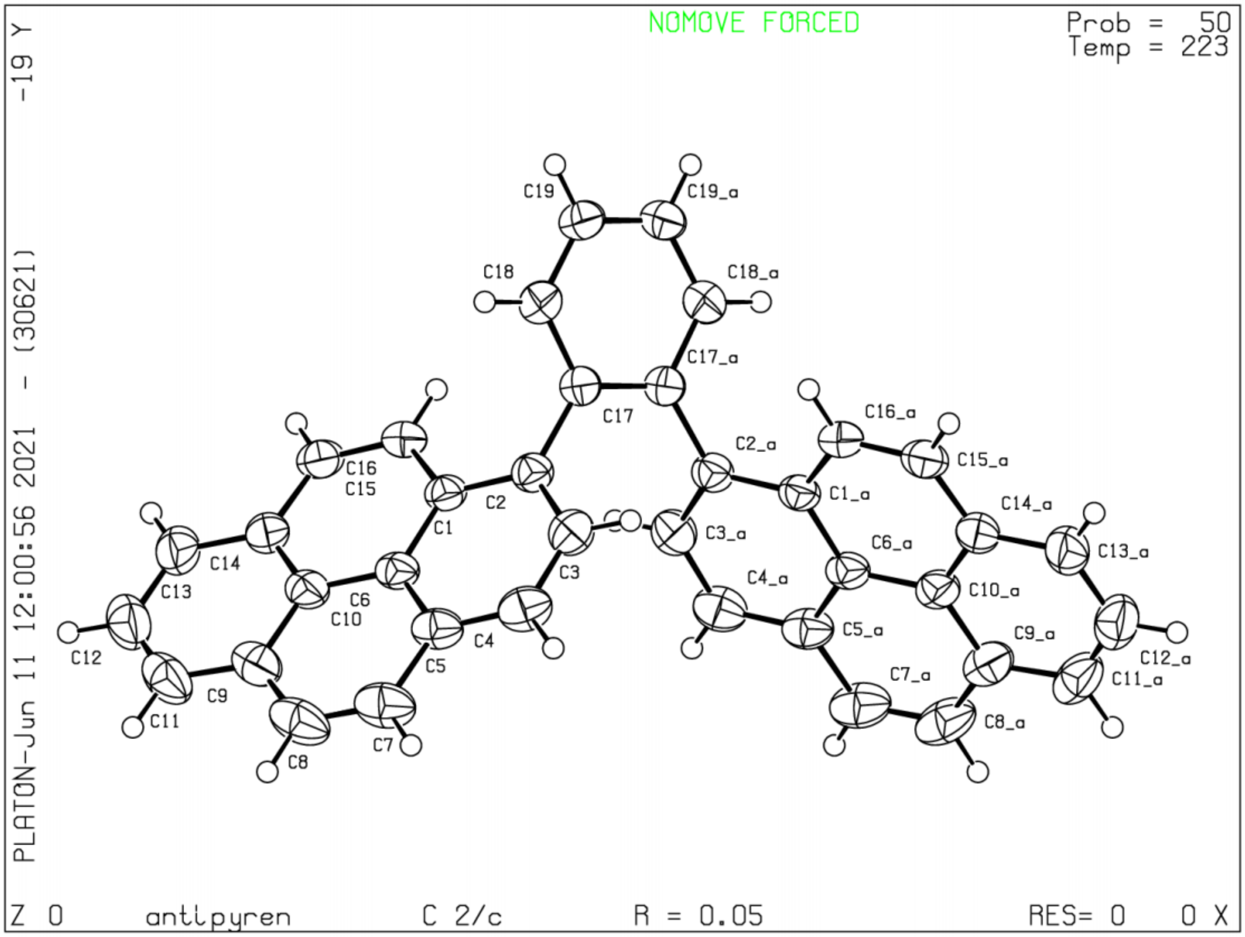

Supplementary Figure S7. Thermal ellipsoid plot of *Anti*-DPyB.

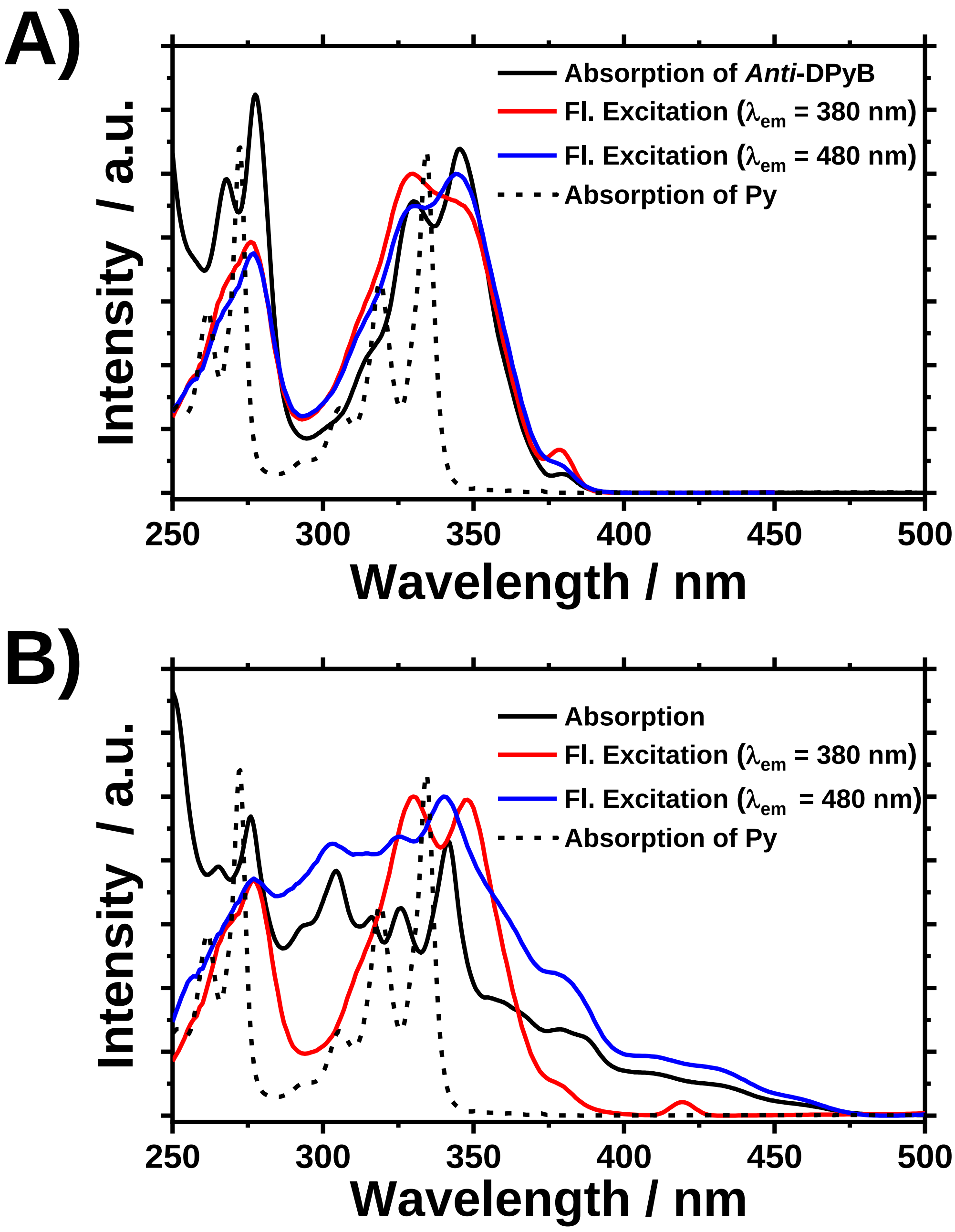

Supplementary Figure S8. Absorption and fluorescence excitation spectra. A) Absorption and fluorescence excitation spectra of *Anti*-DPyB in acetonitrile. B) Absorption and fluorescence excitation spectra of *Syn*-DPyB in acetonitrile.

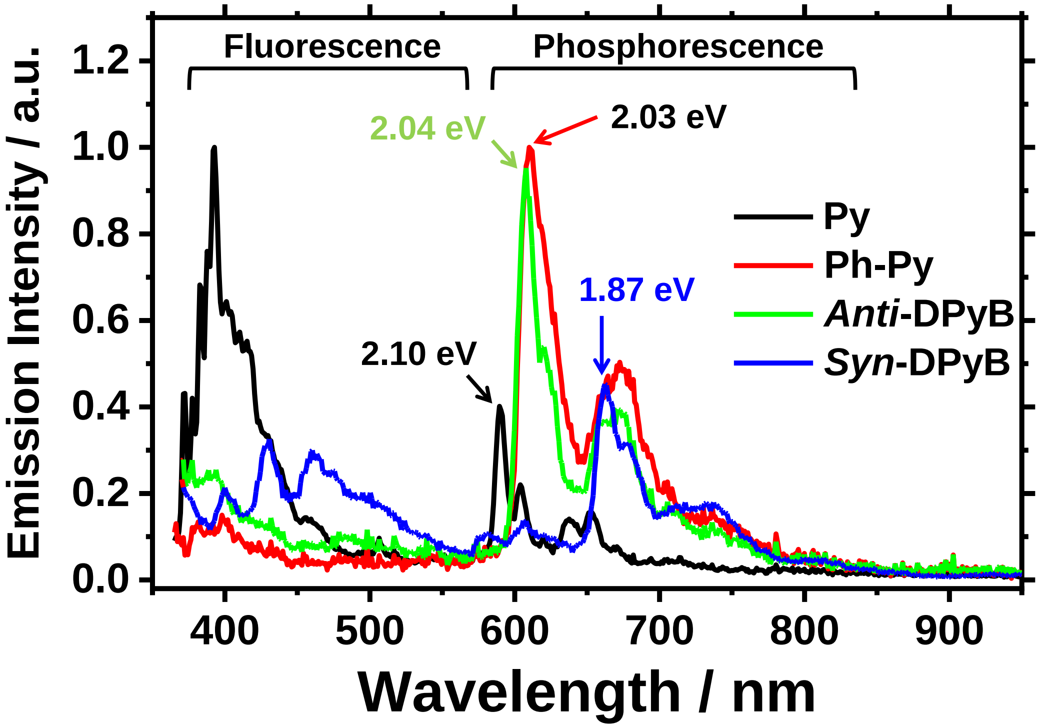

Supplementary Figure S9. Emission spectra of Py, Ph-Py, *Anti*-DPyB, and *Syn*-DPyB measured in MTHF containing iodomethane at 77 K.

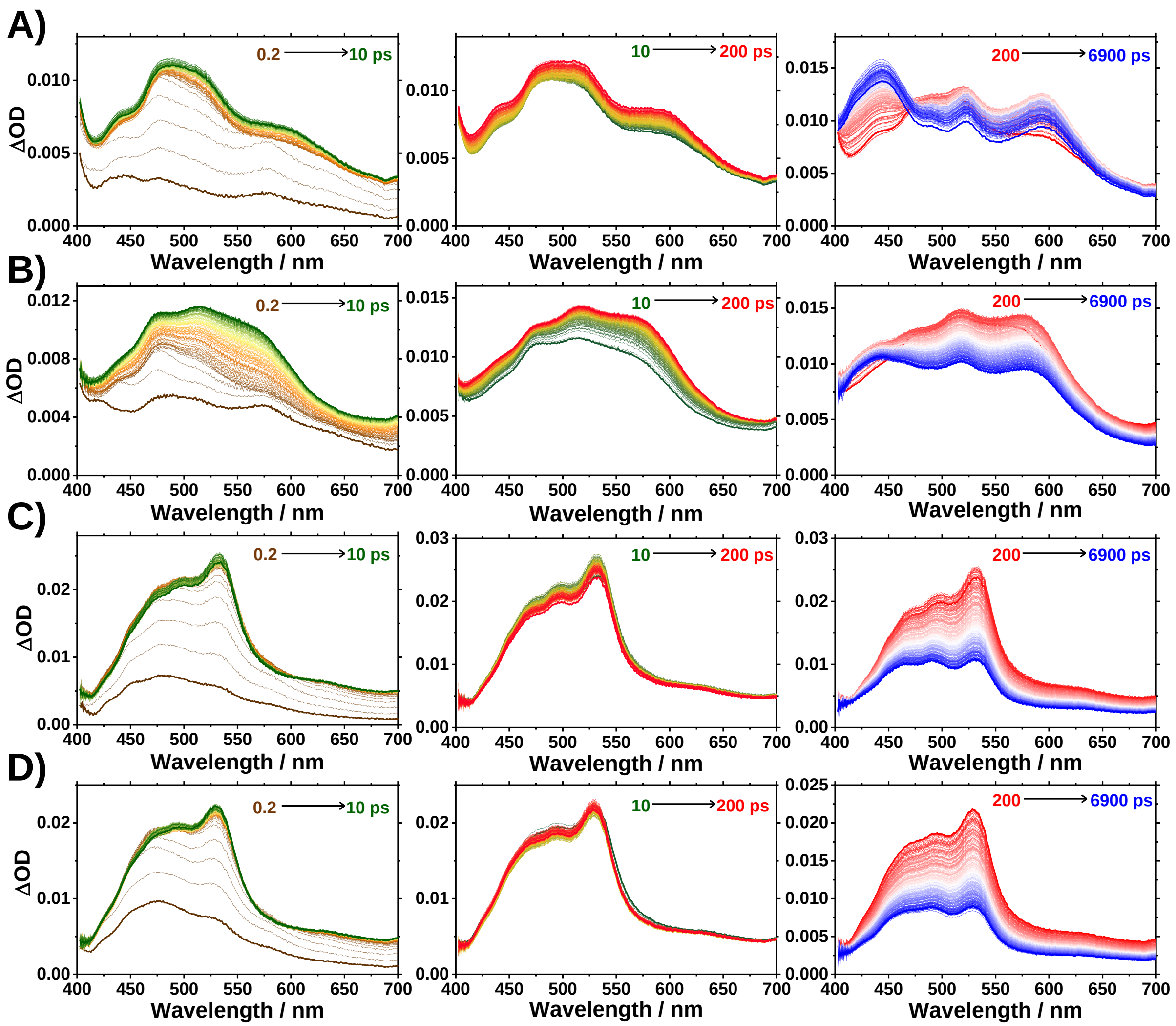

Supplementary Figure S10. Transient absorption spectra. (A, B) Transient absorption spectra of *Anti*-DPyB in *n*-hexane and acetonitrile, respectively. (C, D) Transient absorption spectra of *Syn*-DPyB in *n*-hexane and acetonitrile, respectively.

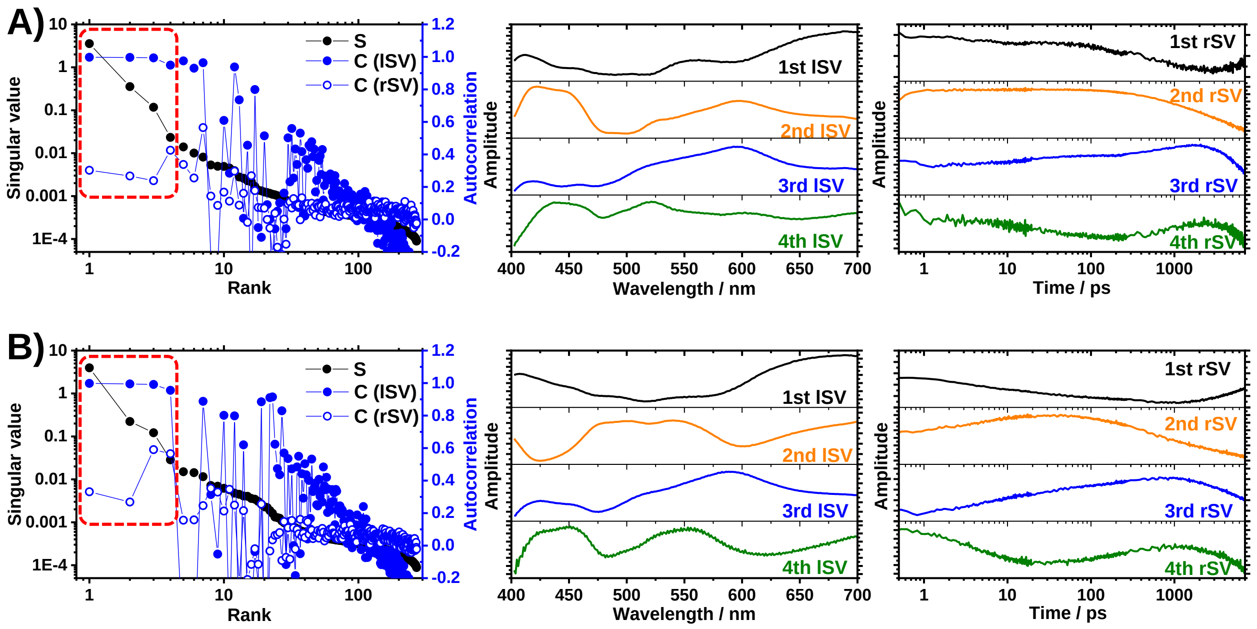

Supplementary Figure S11. Singular value decomposition (SVD) analysis for the TA spectra of *Anti*-DPyB in (A) *n*-hexane and (B) acetonitrile. Singular values and autocorrelations of lSVs and rSVs (left), the first four lSVs (middle), and the first four rSVs (right). Considering rSVs, lSVs, singular values, the number of principal components is considered five.

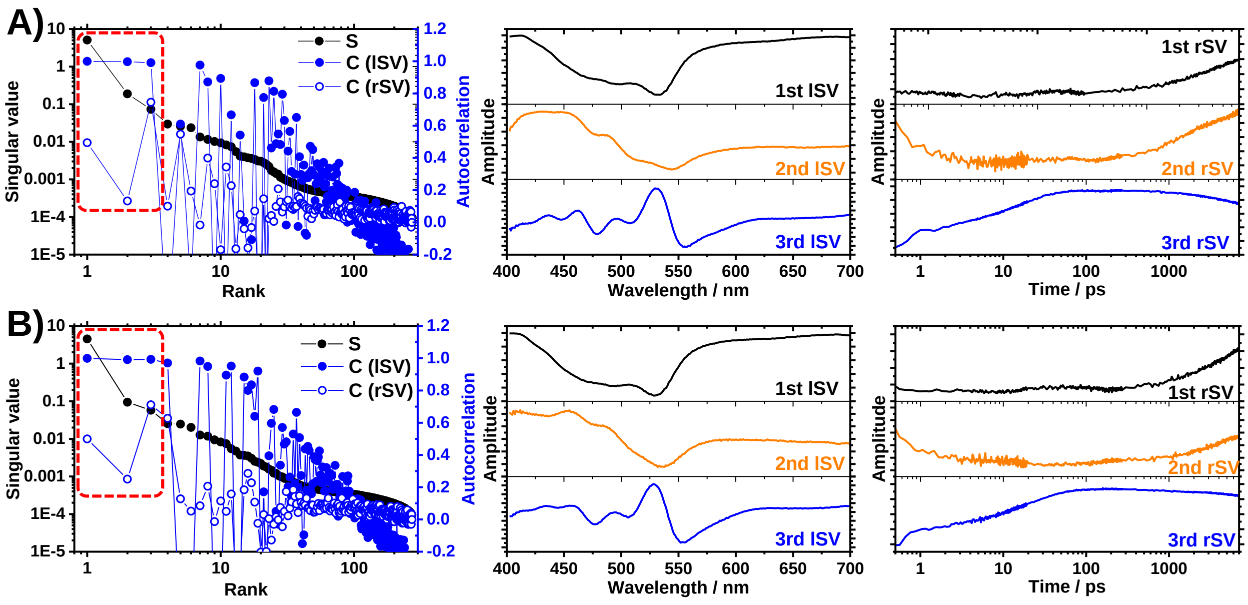

Supplementary Figure S12. Singular value decomposition (SVD) analysis for the TA spectra of *Syn*-DPyB in (A) *n*-hexane and (B) acetonitrile. Singular values and autocorrelations of lSVs and rSVs (left), the first four lSVs (middle), and the first four rSVs (right). Considering rSVs, lSVs, singular values, the number of principal components is considered four.

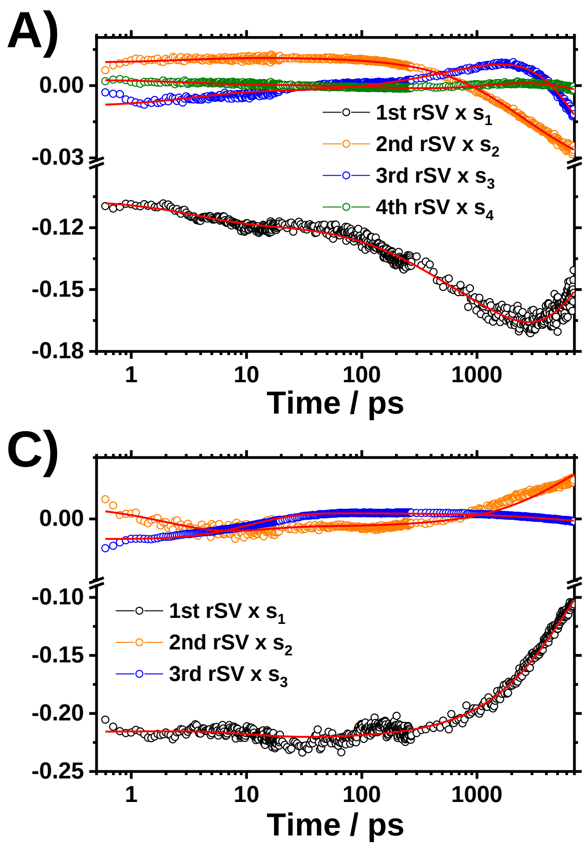

Supplementary Figure S13. Singular values weighted rSVs exponential fitting results for *Anti*-DPyB in (A) *n*-hexane and (B) acetonitrile and *Syn*-DPyB in (C) *n*-hexane and (D) acetonitrile. The Singular values weighted rSVs were globally fitted by exponential functions. The number of exponentials (four exponentials for *Anti*-DPyB, three exponentials for *Syn*-DPyB) and constants are shown in Table 2.

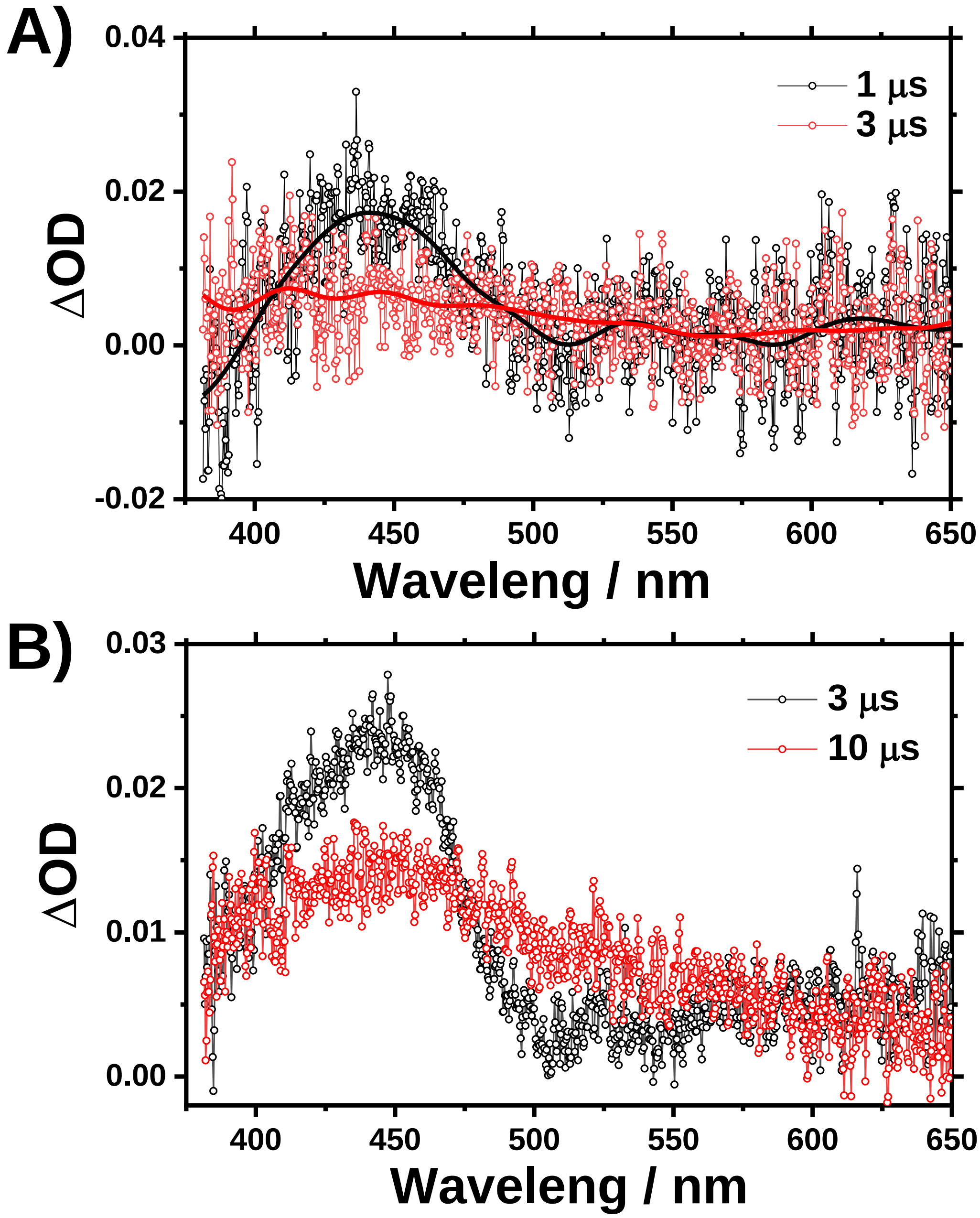

Supplementary Figure S14. Nanosecond transient absorption spectra of *Anti*-DPyB in (A) *n*-hexane and (B) acetonitrile.

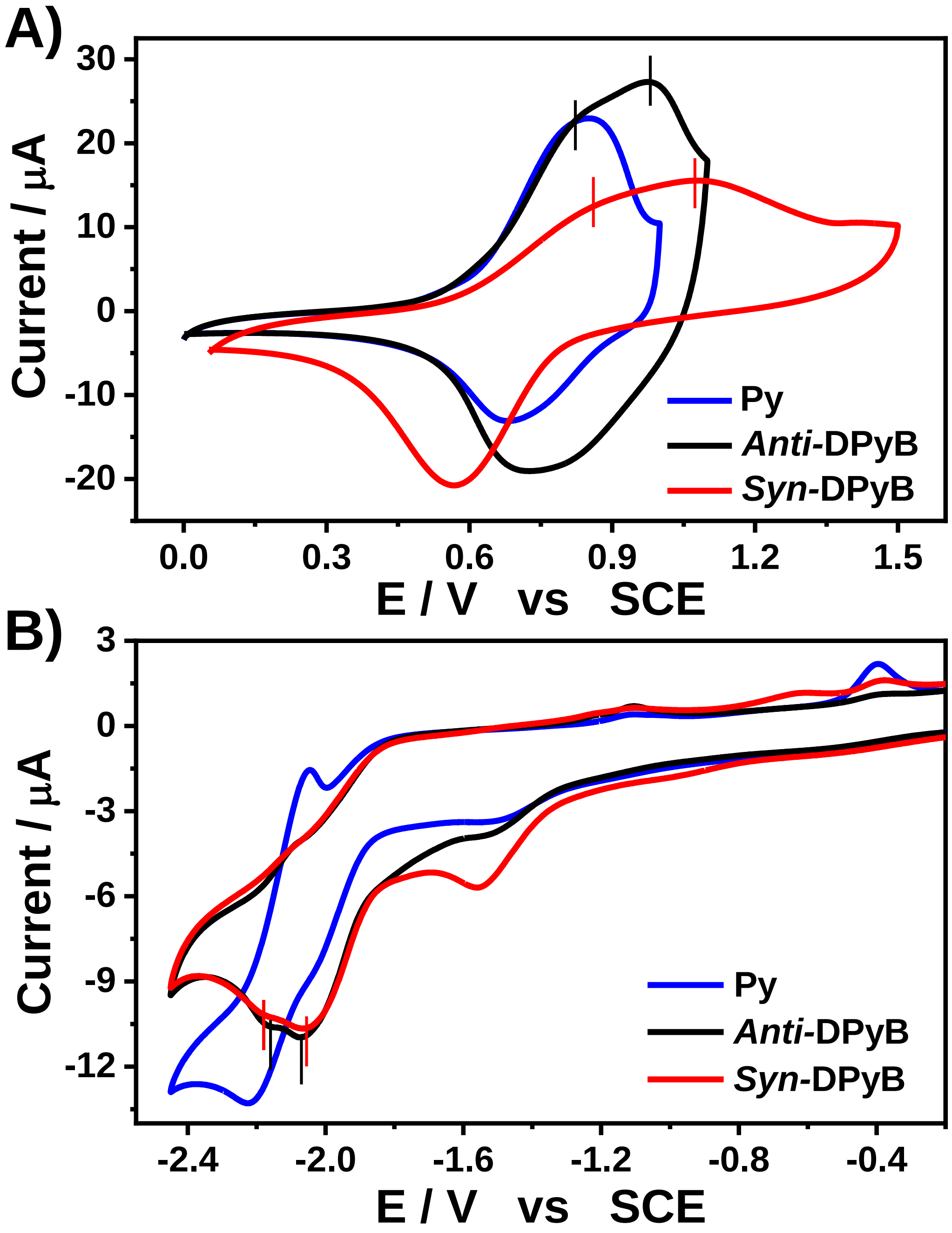

Supplementary Figure S15. Cyclic voltammograms of Py, *Anti*-DPyB, and *Syn*-DPyB. (A) Cyclic voltammograms for the oxidation wave of Py, *Anti*-DPyB, and *Syn*-DPyB in CH_2Cl_2 with 0.1 M $n$$Bu_4NPF_6$ as the supporting electrolyte. Scan rate: 50 mV·s^{-1}. (B) Cyclic voltammograms for the reduction wave of Py, *Anti*-DPyB, and *Syn*-DPyB in THF with 0.1 M $n$$Bu_4NPF_6$ as the supporting electrolyte. Scan rate: 50 mV·s^{-1}.

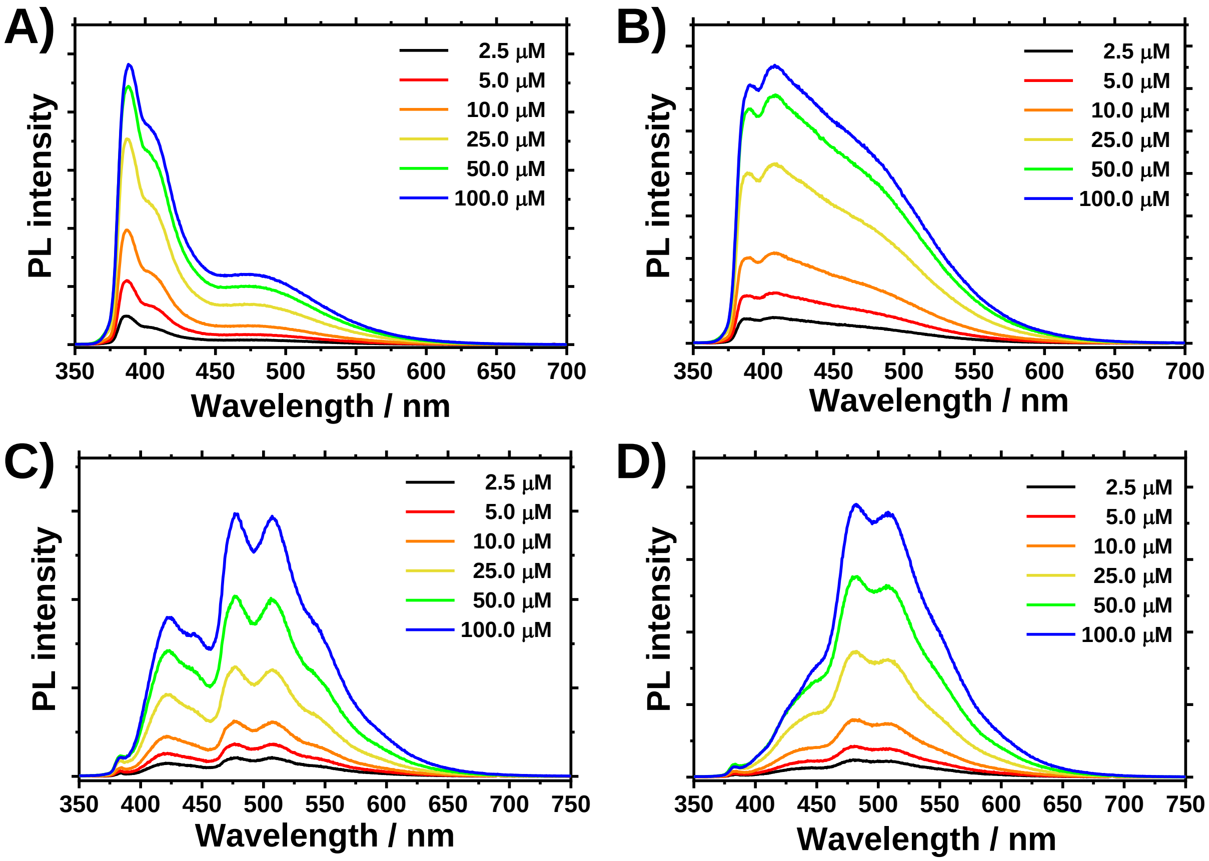

Supplementary Figure S16. Concentration dependence of the emission spectra. Concentration dependence of the emission spectra of *Anti*-DPyB in (A) *n*-hexane and (B) acetonitrile and *Syn*-DPyB in (C) *n*-hexane and (D) acetonitrile.

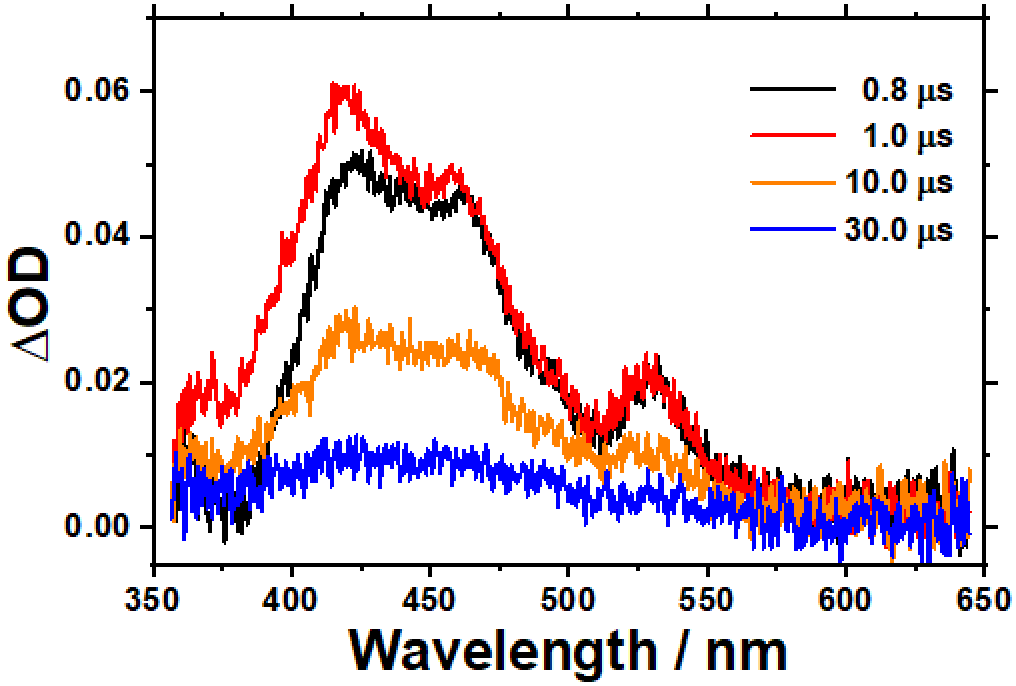

Supplementary Figure S17. Nanosecond transient absorption spectra of 1-(2-bromophenyl)pyrene in dichloromethane (DCM).

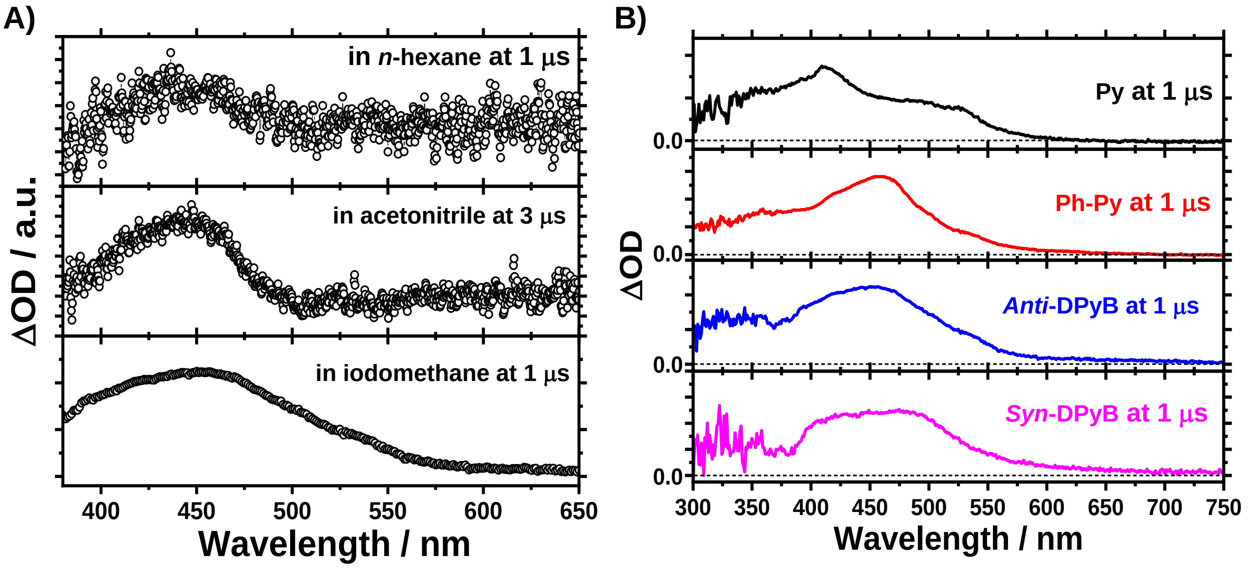

Supplementary Figure S18. Nanosecond transient absorption spectra. (A) Comparison of nanosecond transient absorption spectra of *Anti*-DPyB in *n*-hexane (top), acetonitrile (middle), and iodomethane (bottom). (B) Nanosecond transient absorption spectra of Py (black), Ph-Py (red), *Anti*-DPyB (blue), and *Syn*-DPyB (pink) in iodomethane.

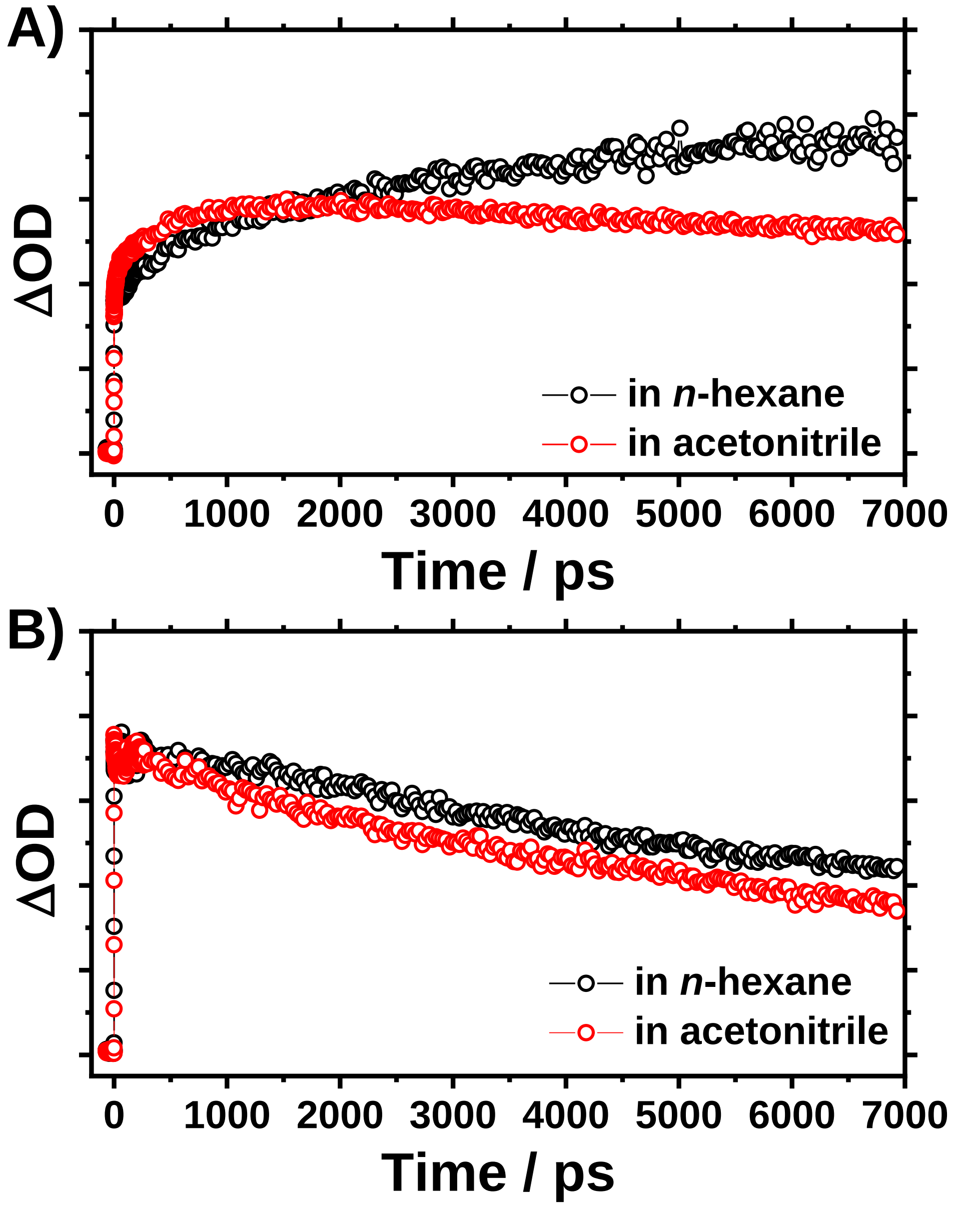

Supplementary Figure S19. Time profiles for transient absorption bands of (A) *Anti*-DpyB and (B) *Syn*-DpyB in *n*-hexane (black) and acetonitrile (red) monitored at 440 nm and 450 nm, respectively.

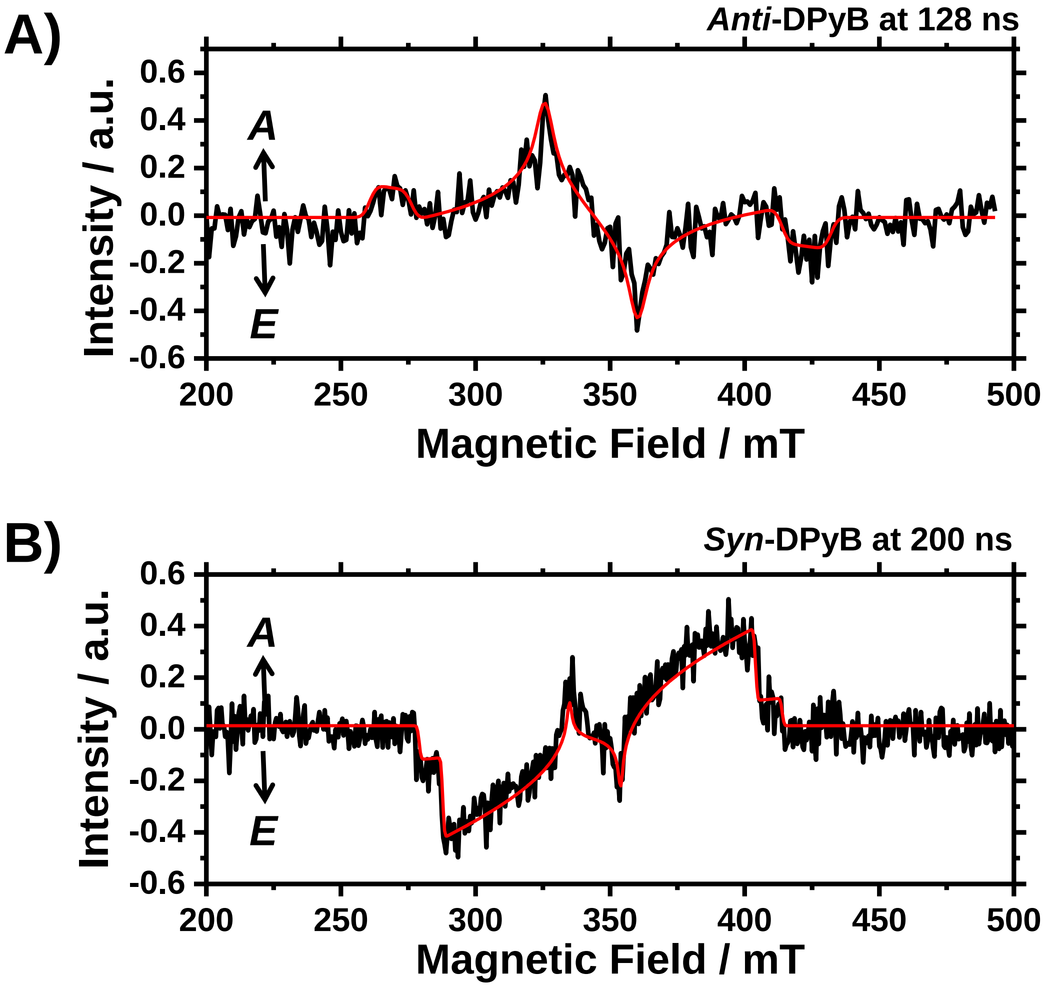

Supplementary Figure S20. X-band (~ 9.728 GHz) perpendicular mode TR-EPR signals of (A) *Anti*-DPyB and (B) *Syn*-DPyB in toluene at 80 K. *A* and *E* indicate absorption and emission, respectively. Simulated spectra of triplets are shown by red lines.

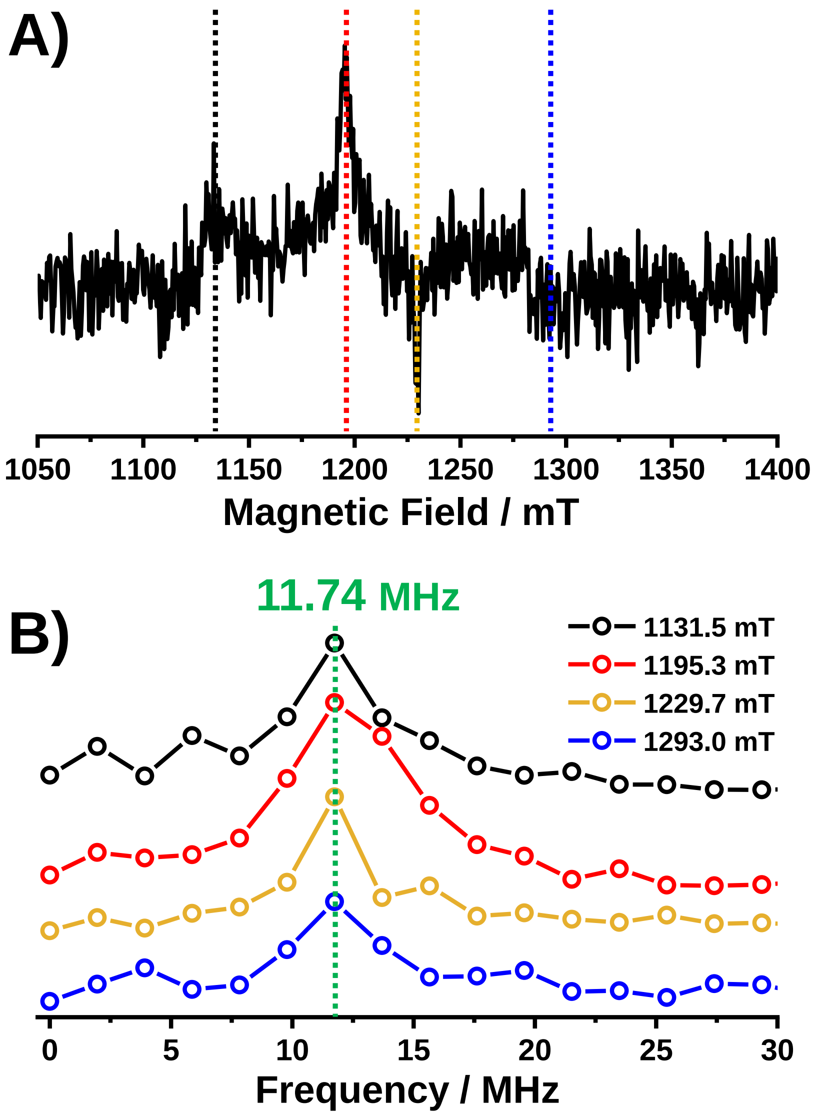

Supplementary Figure S21. (A) Q-band (34 GHz) TR-EPR signal of *Anti*-DPyB. (B) Fourier transforms of nutations measured at 1131.5, 1195.3,1229.7, and 1293.0 mT.

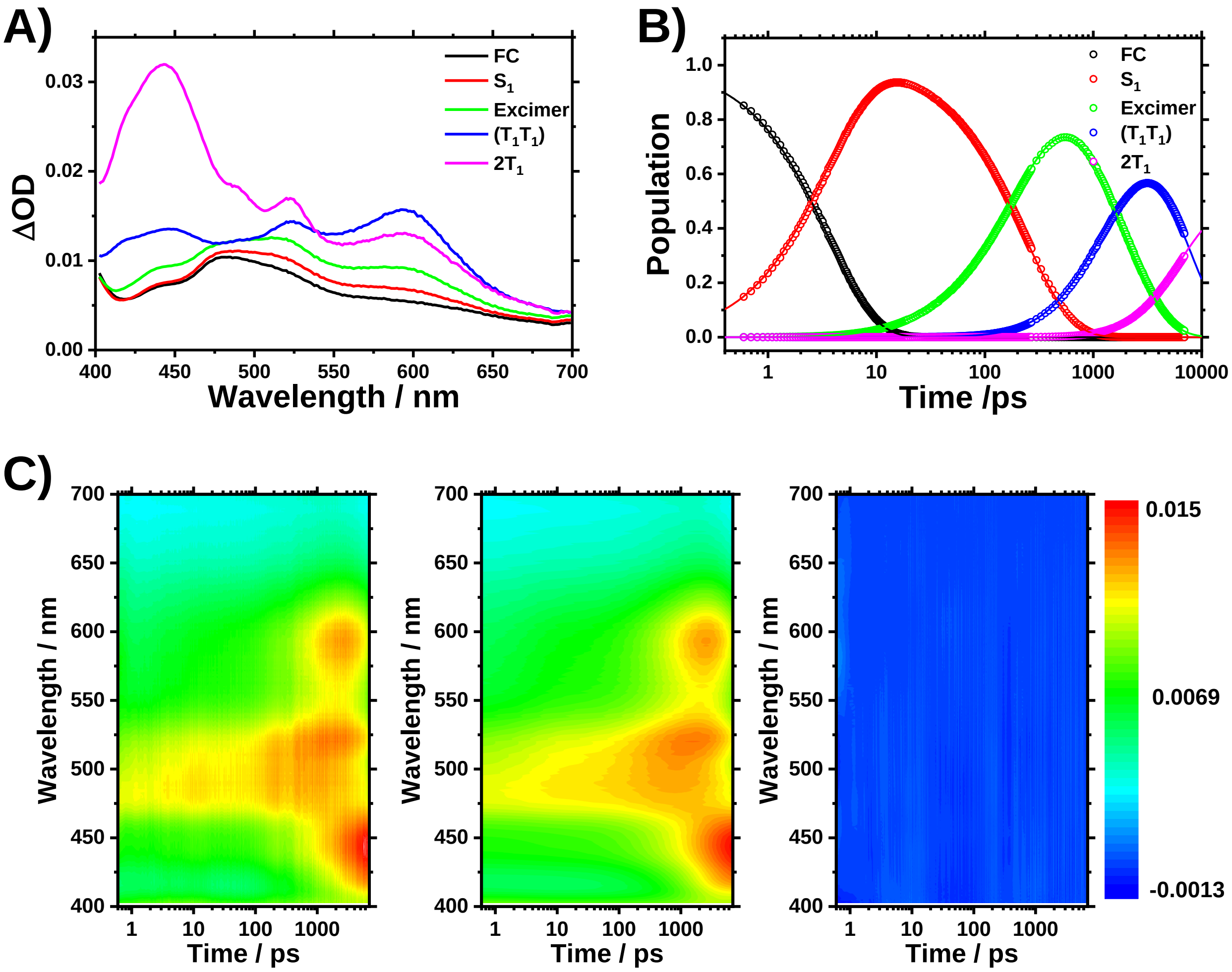

Supplementary Figure S22. TA spectra analysis for *Anti*-DPyB in *n*-hexane with the kinetic model of Kinetic Model (1). (A) Species-associated difference spectra in *n*-hexane. (B) Population changes of intermediates in acetonitrile. The solid lines are the concentrations obtained from the kinetics analysis. The open circles represent the measured time delays. (C) The experimental TA spectra (left), the simulated spectra (middle) by a linear combination of the four SADS curves according to reaction (1), and residuals (right) for *Anti*-DPyB in *n*-hexane.

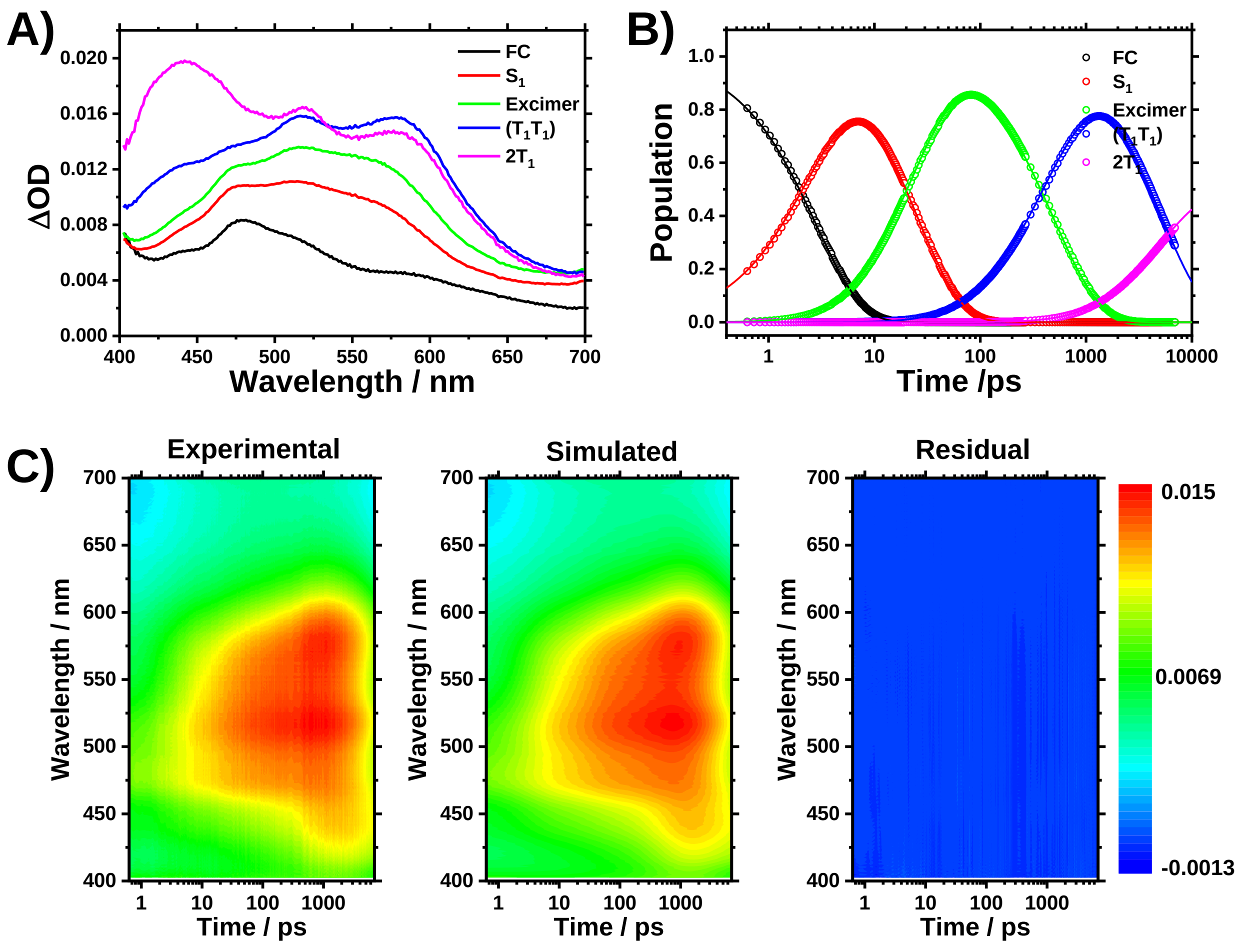

Supplementary Figure S23. TA spectra analysis for *Anti*-DPyB in acetonitrile with the kinetic model of Kinetic Model (1). (A) Species-associated difference spectra in acetonitrile. (B) Population changes of intermediates in acetonitrile. The solid lines are the concentrations obtained from the kinetics analysis. The open circles represent the measured time delays. (C) The experimental TA spectra (left), the simulated spectra (middle) by a linear combination of the four SADS curves according to reaction (S1), and residuals (right) for *Anti*-DPyB in acetonitrile.

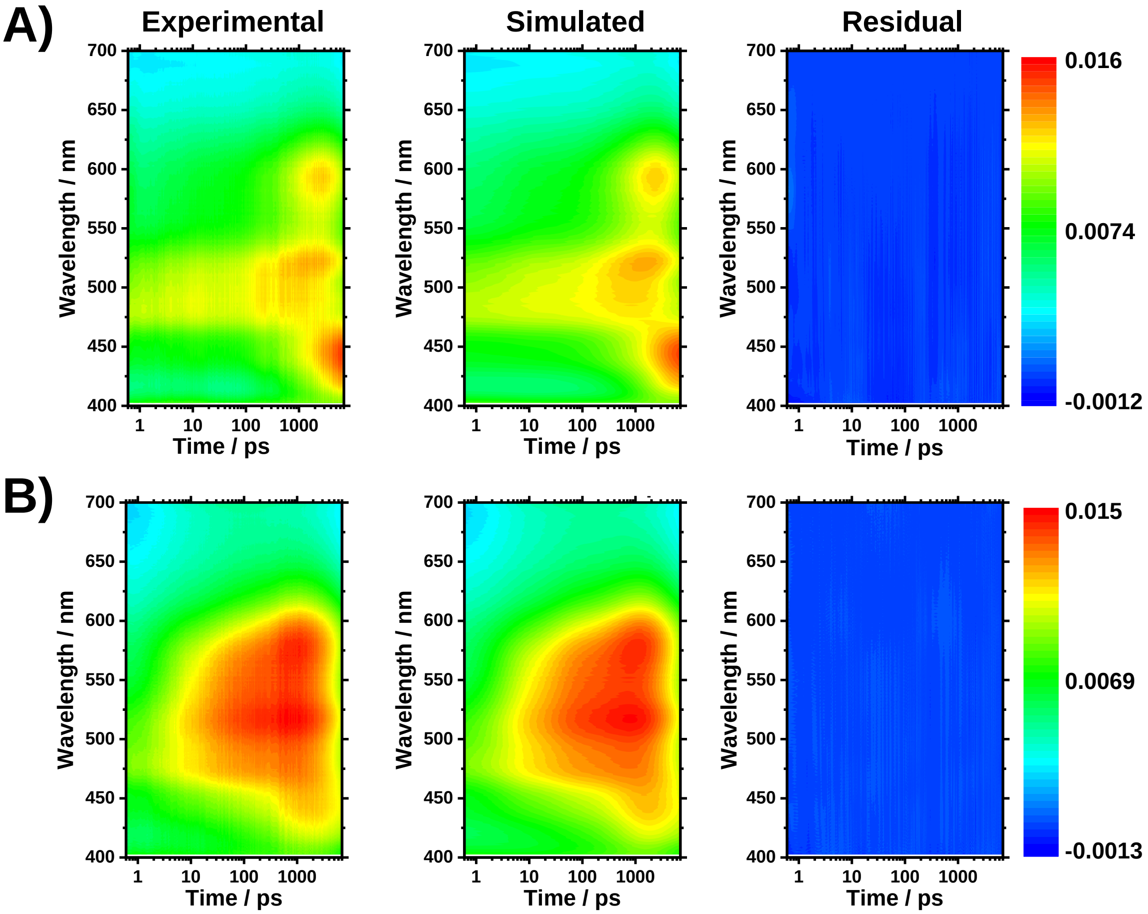

Supplementary Figure S24. TA spectra analysis for *Anti*-DPyB in *n*-hexane and acetonitrile with the kinetic model of Kinetic Model (2). The experimental TA spectra (left), the simulated spectra (middle) by a linear combination of the five SADS curves according to reaction (2), and residuals (right) for *Anti*-DPyB in (A) *n*-hexane and (B) acetonitrile.

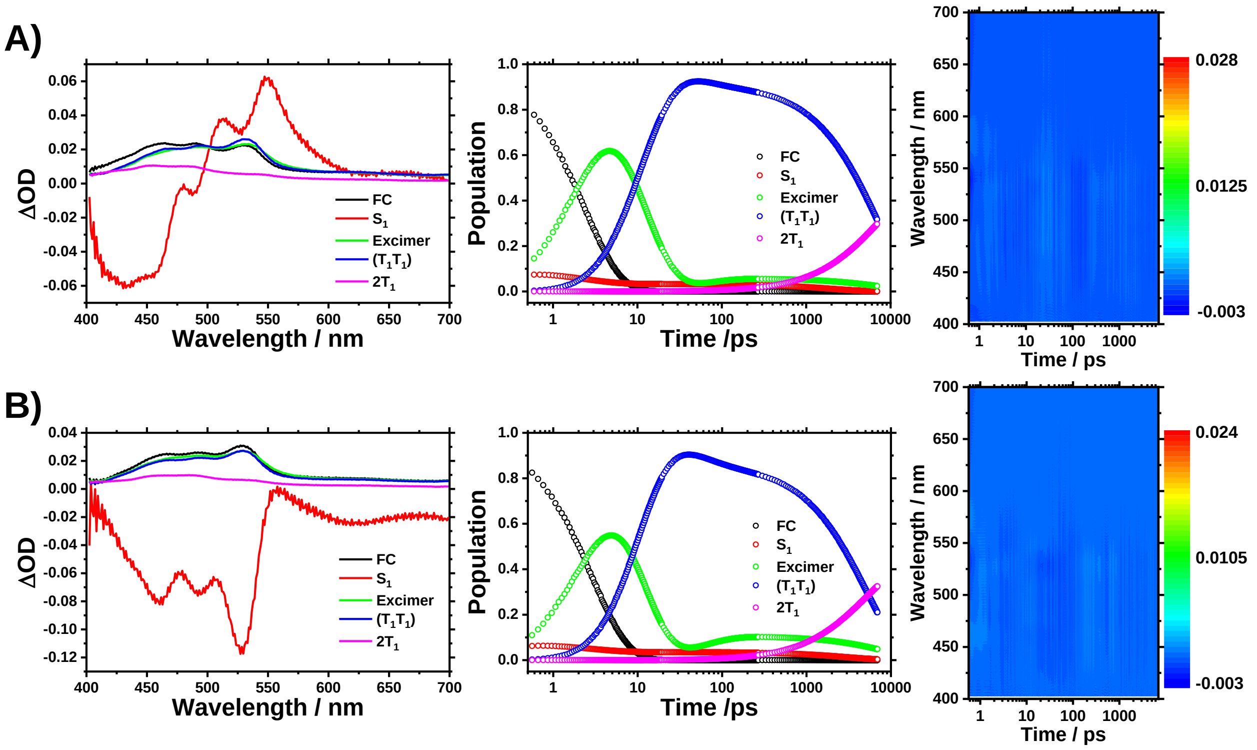

Supplementary Figure S25. TA spectra analysis for *Syn*-DPyB in *n*-hexane and acetonitrile with the kinetic model of Kinetic Model (3). (A) Species-associated difference spectra in *n*-hexane (left), population changes of intermediates in *n*-hexane (middle), and residual (right). (B) Species-associated difference spectra in acetonitrile (left), population changes of intermediates in acetonitrile (middle), and residual (right).

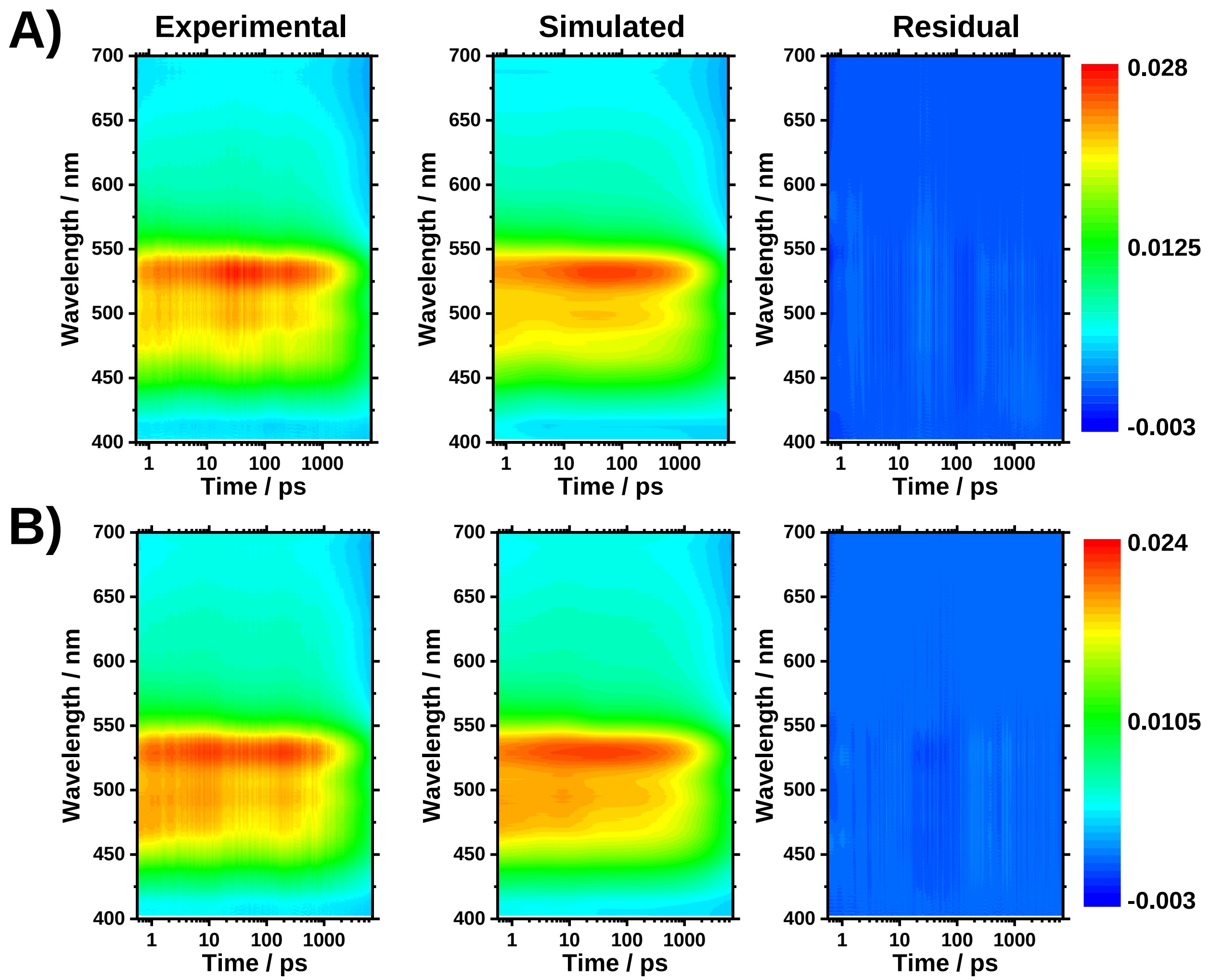

Supplementary Figure S26. TA spectra analysis for *Syn*-DPyB in *n*-hexane and acetonitrile with the kinetic model of Kinetic Model (4). The experimental TA spectra (left), the simulated spectra (middle) by a linear combination of the four SADS curves according to reaction (4), and residuals (right) for *Syn*-DPyB in (A) *n*-hexane and (B) acetonitrile.

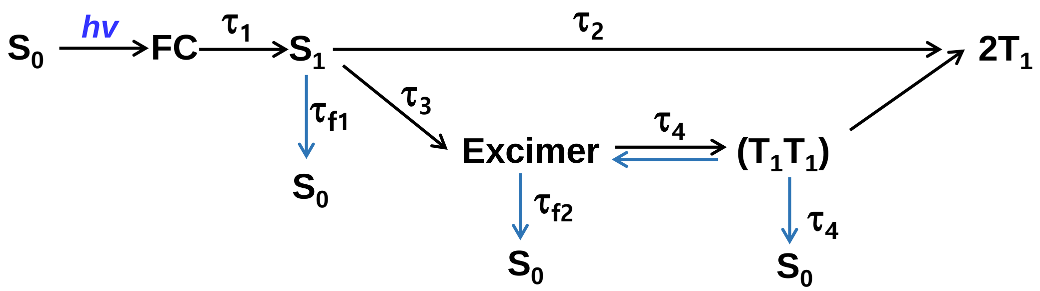

Figure S27. Photoinduced reaction schemes for *Anti*-DPyB containing a direct SF process from the S_1 state to the free triplet state. (S_0: ground state, FC: Franck-Condon state, S_1: singlet excited state, (T_1T_1): correlated triplet pair, and T_1: free triplet state).

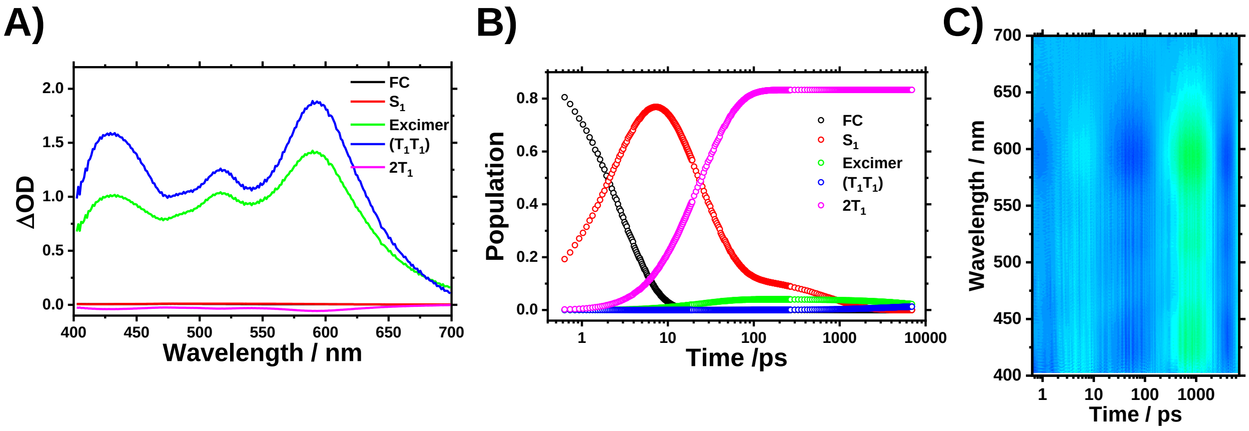

Supplementary Figure S28. TA spectra analysis for *Anti*-DPyB in acetonitrile with the kinetic model of Kinetic Model (Supplementary Figure S27). (A) Species-associated difference spectra in acetonitrile. (B) Population changes of intermediates in acetonitrile. The solid lines are the concentrations obtained from the kinetics analysis. The open circles represent the measured time delays. (C) The residual for *Anti*-DPyB in acetonitrile. The substantial residuals indicate that this kinetic model does not explain the experimental data satisfactorily, unlike Kinetic Model (2).

Supplementary Table S1. Crystal data and structure refinement for *Anti*-DPyB

**Comp.**	***Anti*-DPyB**	
CCDC number	2089494	
Empirical formula	$C_{38}H_{22}$	
Formula weight	478.55	
Temperature	223(2) K	
Wavelength	0.71073 Å	
Crystal system	Monoclinic	
Space group	C_2/c	
Unit cell dimensions	a = 22.365(2) Å	α= 90°
	b = 9.3901(7) Å	β= 115.885(3)°
	c = 12.9994(13) Å	γ= 90°
Volume	2456.1(4) $Å^3$	
Z	4	
Density (calculated)	1.294 Mg/m^3	
Absorption coefficient	0.073 mm^{-1}	
F(000)	1000	
Crystal size	0.320 x 0.240 x 0.120 mm^3	
θ range for data collection	2.688 to 28.506°	
Index ranges	-29<=h<=29, -12<=k<=12, -17<=l<=17	
Reflections collected	40705	
Independent reflections	3076 [R(int) = 0.0644]	
Completeness to θ = 25.242°	99.5 %	
Absorption correction	Semi-empirical from equivalents	
Max. and min. transmission	0.7457 and 0.6261	
Refinement method	Full-matrix least-squares on F^2	
Data / restraints / parameters	3076 / 0 / 172	
Goodness-of-fit on F^2	1.064	
Final R indices [I>2σ(I)]	R_1 = 0.0478, wR_2 = 0.1178	
R indices (all data)	R_1 = 0.0727, wR_2 = 0.1377	
Extinction coefficient	n/a	
Largest diff. peak and hole	0.169 and -0.187 e. $Å^{-3}$	

Supplementary Table S2. Atomic coordinates (x 10^4) and equivalent isotropic displacement parameters (Å 2 x 10^3) for *Anti*-DPyB. U(eq) is defined as one third of the trace of the orthogonalized Uij tensor

	**X**	**Y**	**Z**	**U(eq)**
C(1)	3734(1)	5147(1)	1909(1)	36(1)
C(2)	4392(1)	5463(1)	2694(1)	38(1)
C(3)	4729(1)	4530(2)	3597(1)	46(1)
C(4)	4423(1)	3340(2)	3770(1)	51(1)
C(5)	3763(1)	3031(2)	3048(1)	45(1)
C(6)	3414(1)	3935(1)	2100(1)	38(1)
C(7)	3413(1)	1837(2)	3221(2)	60(1)
C(8)	2772(1)	1589(2)	2521(2)	63(1)
C(9)	2403(1)	2483(2)	1567(2)	51(1)
C(10)	2735(1)	3645(2)	1345(1)	41(1)
C(11)	1728(1)	2259(2)	836(2)	64(1)
C(12)	1393(1)	3135(2)	-84(2)	65(1)
C(13)	1716(1)	4249(2)	-316(2)	56(1)
C(14)	2386(1)	4522(2)	383(1)	44(1)
C(15)	2737(1)	5671(2)	172(1)	47(1)
C(16)	3370(1)	5978(2)	897(1)	42(1)
C(17)	4716(1)	6810(2)	2594(1)	39(1)
C(18)	4450(1)	8114(2)	2714(2)	50(1)
C(19)	4728(1)	9390(2)	2619(2)	56(1)

Supplementary Table S3. Bond lengths [Å] and angles [°] for *Anti*-DPyB

C(1)-C(2)	1.4094(19)
C(1)-C(6)	1.4225(19)
C(1)-C(16)	1.438(2)
C(2)-C(3)	1.393(2)
C(2)-C(17)	1.4914(19)
C(3)-C(4)	1.380(2)
C(3)-H(3)	0.9400
C(4)-C(5)	1.391(2)
C(4)-H(4)	0.9400
C(5)-C(6)	1.418(2)
C(5)-C(7)	1.439(2)
C(6)-C(10)	1.428(2)
C(7)-C(8)	1.342(3)
C(7)-H(7)	0.9400
C(8)-C(9)	1.424(3)
C(8)-H(8)	0.9400
C(9)-C(11)	1.404(3)
C(9)-C(10)	1.419(2)
C(10)-C(14)	1.414(2)
C(11)-C(12)	1.373(3)
C(11)-H(11)	0.9400
C(12)-C(13)	1.377(3)
C(12)-H(12)	0.9400
C(13)-C(14)	1.396(2)
C(13)-H(13)	0.9400
C(14)-C(15)	1.429(2)
C(15)-C(16)	1.346(2)
C(15)-H(15)	0.9400
C(16)-H(16)	0.9400
C(17)-C(17)#1	1.396(3)
C(17)-C(18)	1.399(2)
C(18)-C(19)	1.381(2)
C(18)-H(18)	0.9400
C(19)-C(19)#1	1.378(4)
C(19)-H(19)	0.9400
C(2)-C(1)-C(6)	119.25(13)
C(2)-C(1)-C(16)	122.87(13)
C(6)-C(1)-C(16)	117.87(13)
C(3)-C(2)-C(1)	119.18(13)
C(3)-C(2)-C(17)	120.17(13)
C(1)-C(2)-C(17)	120.59(12)
C(4)-C(3)-C(2)	121.50(15)
C(4)-C(3)-H(3)	119.3
C(2)-C(3)-H(3)	119.3
C(3)-C(4)-C(5)	121.02(15)
C(3)-C(4)-H(4)	119.5
C(5)-C(4)-H(4)	119.5
C(4)-C(5)-C(6)	118.76(14)

C(4)-C(5)-C(7)	123.10(15)
C(6)-C(5)-C(7)	118.13(15)
C(5)-C(6)-C(1)	120.16(13)
C(5)-C(6)-C(10)	120.00(13)
C(1)-C(6)-C(10)	119.83(13)
C(8)-C(7)-C(5)	121.49(17)
C(8)-C(7)-H(7)	119.3
C(5)-C(7)-H(7)	119.3
C(7)-C(8)-C(9)	121.97(16)
C(7)-C(8)-H(8)	119.0
C(9)-C(8)-H(8)	119.0
C(11)-C(9)-C(10)	118.72(18)
C(11)-C(9)-C(8)	122.96(16)
C(10)-C(9)-C(8)	118.32(16)
C(14)-C(10)-C(9)	119.52(15)
C(14)-C(10)-C(6)	120.46(13)
C(9)-C(10)-C(6)	120.01(15)
C(12)-C(11)-C(9)	121.11(17)
C(12)-C(11)-H(11)	119.4
C(9)-C(11)-H(11)	119.4
C(11)-C(12)-C(13)	120.43(18)
C(11)-C(12)-H(12)	119.8
C(13)-C(12)-H(12)	119.8
C(12)-C(13)-C(14)	120.93(19)
C(12)-C(13)-H(13)	119.5
C(14)-C(13)-H(13)	119.5
C(13)-C(14)-C(10)	119.27(15)
C(13)-C(14)-C(15)	122.52(16)
C(10)-C(14)-C(15)	118.20(14)
C(16)-C(15)-C(14)	121.70(15)
C(16)-C(15)-H(15)	119.1
C(14)-C(15)-H(15)	119.1
C(15)-C(16)-C(1)	121.71(14)
C(15)-C(16)-H(16)	119.1
C(1)-C(16)-H(16)	119.1
C(17)#1-C(17)-C(18)	118.93(9)
C(17)#1-C(17)-C(2)	121.93(7)
C(18)-C(17)-C(2)	119.13(13)
C(19)-C(18)-C(17)	121.26(15)
C(19)-C(18)-H(18)	119.4
C(17)-C(18)-H(18)	119.4
C(19)#1-C(19)-C(18)	119.76(10)
C(19)#1-C(19)-H(19)	120.1

Symmetry transformations used to generate equivalent atoms:
#1 -x+1, y, -z+1/2

Supplementary Table S4. Anisotropic displacement parameters (Å 2 x 10^3) for *Anti*-DPyB. The anisotropic displacement factor exponent takes the form: $-2\pi^2[h^2a^{*2}U^{11} + ... + 2hka^*b^*U^{12}]$

	**U11**	**U22**	**U33**	**U23**	**U13**	**U12**
C(1)	41(1)	29(1)	43(1)	-1(1)	22(1)	2(1)
C(2)	41(1)	34(1)	42(1)	-1(1)	22(1)	1(1)
C(3)	46(1)	47(1)	44(1)	2(1)	18(1)	4(1)
C(4)	65(1)	46(1)	43(1)	11(1)	26(1)	7(1)
C(5)	62(1)	37(1)	47(1)	3(1)	34(1)	0(1)
C(6)	46(1)	31(1)	45(1)	-3(1)	28(1)	0(1)
C(7)	87(1)	46(1)	60(1)	9(1)	46(1)	-5(1)
C(8)	87(1)	51(1)	73(1)	-4(1)	55(1)	-22(1)
C(9)	61(1)	44(1)	66(1)	-15(1)	44(1)	-13(1)
C(10)	47(1)	34(1)	54(1)	-12(1)	32(1)	-4(1)
C(11)	63(1)	60(1)	90(1)	-28(1)	54(1)	-24(1)
C(12)	47(1)	68(1)	87(1)	-30(1)	36(1)	-12(1)
C(13)	43(1)	54(1)	67(1)	-18(1)	21(1)	0(1)
C(14)	43(1)	37(1)	54(1)	-12(1)	23(1)	1(1)
C(15)	49(1)	33(1)	51(1)	-1(1)	15(1)	4(1)
C(16)	46(1)	29(1)	50(1)	2(1)	20(1)	0(1)
C(17)	37(1)	35(1)	43(1)	0(1)	14(1)	0(1)
C(18)	42(1)	41(1)	64(1)	-5(1)	22(1)	3(1)
C(19)	45(1)	34(1)	74(1)	-5(1)	13(1)	4(1)

Supplementary Table S5. Hydrogen coordinates (x 10^4) and isotropic displacement parameters (Å 2 x 10^3) for *Anti*-DPyB

	x	y	z	U(eq)
H(3)	5175	4716	4100	55
H(4)	4664	2729	4386	61
H(7)	3639	1217	3837	71
H(8)	2559	803	2664	76
H(11)	1502	1497	979	76
H(12)	940	2974	-558	78
H(13)	1482	4834	-953	67
H(15)	2520	6227	-490	56
H(16)	3581	6757	737	50
H(18)	4075	8121	2863	60
H(19)	4548	10256	2716	67

Supplementary Table S6. Torsion angles [°] for *Anti*-DpyB

C(6)-C(1)-C(2)-C(3)	4.1(2)
C(16)-C(1)-C(2)-C(3)	-174.22(13)
C(6)-C(1)-C(2)-C(17)	-173.05(12)
C(16)-C(1)-C(2)-C(17)	8.6(2)
C(1)-C(2)-C(3)-C(4)	-3.0(2)
C(17)-C(2)-C(3)-C(4)	174.17(14)
C(2)-C(3)-C(4)-C(5)	-0.3(2)
C(3)-C(4)-C(5)-C(6)	2.3(2)
C(3)-C(4)-C(5)-C(7)	-177.33(15)
C(4)-C(5)-C(6)-C(1)	-1.1(2)
C(7)-C(5)-C(6)-C(1)	178.56(13)
C(4)-C(5)-C(6)-C(10)	-179.89(13)
C(7)-C(5)-C(6)-C(10)	-0.2(2)
C(2)-C(1)-C(6)-C(5)	-2.1(2)
C(16)-C(1)-C(6)-C(5)	176.31(13)
C(2)-C(1)-C(6)-C(10)	176.69(12)
C(16)-C(1)-C(6)-C(10)	-4.88(19)
C(4)-C(5)-C(7)-C(8)	178.47(17)
C(6)-C(5)-C(7)-C(8)	-1.2(2)
C(5)-C(7)-C(8)-C(9)	0.5(3)
C(7)-C(8)-C(9)-C(11)	-178.80(17)
C(7)-C(8)-C(9)-C(10)	1.6(3)
C(11)-C(9)-C(10)-C(14)	-1.6(2)
C(8)-C(9)-C(10)-C(14)	178.05(14)
C(11)-C(9)-C(10)-C(6)	177.42(13)
C(8)-C(9)-C(10)-C(6)	-3.0(2)
C(5)-C(6)-C(10)-C(14)	-178.72(13)
C(1)-C(6)-C(10)-C(14)	2.5(2)
C(5)-C(6)-C(10)-C(9)	2.3(2)
C(1)-C(6)-C(10)-C(9)	-176.49(13)
C(10)-C(9)-C(11)-C(12)	0.5(2)
C(8)-C(9)-C(11)-C(12)	-179.11(17)
C(9)-C(11)-C(12)-C(13)	0.7(3)
C(11)-C(12)-C(13)-C(14)	-0.7(3)
C(12)-C(13)-C(14)-C(10)	-0.4(2)
C(12)-C(13)-C(14)-C(15)	-179.73(15)
C(9)-C(10)-C(14)-C(13)	1.5(2)
C(6)-C(10)-C(14)-C(13)	-177.46(13)
C(9)-C(10)-C(14)-C(15)	-179.10(13)
C(6)-C(10)-C(14)-C(15)	1.9(2)
C(13)-C(14)-C(15)-C(16)	175.41(15)
C(10)-C(14)-C(15)-C(16)	-4.0(2)
C(14)-C(15)-C(16)-C(1)	1.5(2)
C(2)-C(1)-C(16)-C(15)	-178.65(14)
C(6)-C(1)-C(16)-C(15)	3.0(2)
C(3)-C(2)-C(17)-C(17)#1	64.4(2)
C(1)-C(2)-C(17)-C(17)#1	-118.42(19)
C(3)-C(2)-C(17)-C(18)	-114.31(17)
C(1)-C(2)-C(17)-C(18)	62.83(19)
C(17)#1-C(17)-C(18)-C(19)	1.8(3)

C(2)-C(17)-C(18)-C(19)	-179.44(16)
C(17)-C(18)-C(19)-C(19)#1	1.2(3)

Symmetry transformations used to generate equivalent atoms:
#1 -x+1, y, -z+1/2

Supplementary references

1. SMART and SAINT; Bruker Analytical X-ray Division: Madison, WI, (2002).

2. Sheldrick GM. SHELXTL-PLUS Software Package; Bruker Analytical X-ray Division: Madison, WI, (2002).

3. Bensasson R, Land EJ. Triplet-triplet extinction coefficients via energy transfer. *Trans. Faraday Soc.* **67**, 1904-1915 (1971).

4. Stoll S, Schweiger A. EasySpin, a comprehensive software package for spectral simulation and analysis in EPR. *J Magn Reson* **178**, 42-55 (2006).

5. Fuemmeler EG, *et al.* A Direct Mechanism of Ultrafast Intramolecular Singlet Fission in Pentacene Dimers. *ACS Central Sci.* **2**, 316-324 (2016).

6. Fumanal M, Corminboeuf C. Direct, Mediated, and Delayed Intramolecular Singlet Fission Mechanism in Donor-Acceptor Copolymers. *J. Phys. Chem. Lett.* **11**, 9788-9794 (2020).

7. Dover CB, *et al.* Endothermic Singlet Fission is Hindered by Excimer Formation. *Nat. Chem.* **10**, 305-310 (2018).
