# Supplementary material for: Singlet fission dynamics modulated by molecular configuration in covalently linked pyrene dimers, Anti- and Syn-1,2-di(pyrenyl)benzene
Source: Commun Chem. 2023 Jan 17;6:16. doi: 10.1038/s42004-023-00816-6 (PMC9845327; doi:10.1038/s42004-023-00816-6)
Supplement: Supplementary file 3 — Description of Additional Supplementary Files [file 42004_2023_816_MOESM3_ESM.pdf]

Description of Additional Supplementary Files

File name: Supplementary Data 1

Description: cif file of Anti-DpyB

File name: Supplementary Data 2

Description: Cartesian coordinates from computational studies
