# Supplementary material for: Singlet fission dynamics modulated by molecular configuration in covalently linked pyrene dimers, Anti- and Syn-1,2-di(pyrenyl)benzene
Source: Commun Chem. 2023 Jan 17;6:16. doi: 10.1038/s42004-023-00816-6 (PMC9845327; doi:10.1038/s42004-023-00816-6)
Supplement: Supplementary file 5 — Supplementary Data 2 [file 42004_2023_816_MOESM5_ESM.docx]

**Supplementary Data 2.1** **|** Cartesian coordinates for the structure of *Anti*-DPyB optimized by using B3LYP/6-31G(d,p)

Symbolic Z-matrix:

Charge = 0 Multiplicity = 1

| Atom | X | Y | Z | Atom | X | Y | Z |
| --- | --- | --- | --- | --- | --- | --- | --- |
| C | -0.629937 | 4.803254 | -0.299418 | C | 5.112692 | -0.097737 | -0.905796 |
| C | 0.629872 | 4.803278 | 0.299377 | C | 4.484682 | -0.871433 | 0.116711 |
| C | 1.250479 | 3.592368 | 0.595996 | C | 5.127708 | -2.051798 | 0.603155 |
| C | 0.638425 | 2.361351 | 0.304824 | C | 4.475803 | -2.82169 | 1.626053 |
| C | -0.638431 | 2.361319 | -0.304879 | C | 3.267792 | -2.446029 | 2.127455 |
| C | -1.250514 | 3.592331 | -0.596037 | C | 6.356057 | -0.509174 | -1.412485 |
| C | 1.326167 | 1.097532 | 0.711344 | C | 6.976902 | -1.658533 | -0.928379 |
| C | -1.326144 | 1.097495 | -0.711374 | C | 6.371459 | -2.422766 | 0.067139 |
| C | 2.579607 | 0.717534 | 0.168736 | H | -1.124962 | 5.739323 | -0.540691 |
| C | 3.220197 | -0.470346 | 0.64777 | H | 1.124858 | 5.739364 | 0.540662 |
| C | 2.598794 | -1.264649 | 1.658479 | H | 2.223145 | 3.588376 | 1.078973 |
| C | 1.351172 | -0.86412 | 2.158929 | H | -2.223185 | 3.588322 | -1.079006 |
| C | 0.73387 | 0.288988 | 1.691694 | H | 0.868915 | -1.463588 | 2.926293 |
| C | -0.733826 | 0.288884 | -1.691657 | H | -0.227591 | 0.58313 | 2.100663 |
| C | -1.35112 | -0.864243 | -2.158843 | H | 0.227649 | 0.582996 | -2.100615 |
| C | -2.598763 | -1.264743 | -1.658413 | H | -0.868824 | -1.463772 | -2.926135 |
| C | -3.220184 | -0.470386 | -0.647759 | H | -4.905769 | 1.653198 | 2.175788 |
| C | -2.5796 | 0.717518 | -0.168772 | H | -2.746161 | 2.348648 | 1.263431 |
| C | -4.484681 | -0.871449 | -0.116708 | H | -2.782978 | -3.038878 | -2.898561 |
| C | -5.112706 | -0.097719 | 0.905767 | H | -4.969916 | -3.718102 | -1.991345 |
| C | -4.436558 | 1.074046 | 1.384664 | H | -6.85975 | -3.319611 | -0.438899 |
| C | -3.234248 | 1.461494 | 0.876263 | H | -7.938964 | -1.962023 | 1.330766 |
| C | -3.267756 | -2.446135 | -2.127361 | H | -6.830988 | 0.081698 | 2.191148 |
| C | -4.47579 | -2.821759 | -1.625983 | H | 2.746148 | 2.348625 | -1.263513 |
| C | -5.127708 | -2.051821 | -0.603129 | H | 4.905767 | 1.653157 | -2.175853 |
| C | -6.371478 | -2.422758 | -0.06713 | H | 4.969925 | -3.718029 | 1.99143 |
| C | -6.976938 | -1.658488 | 0.928347 | H | 2.783038 | -3.03873 | 2.898702 |
| C | -6.356085 | -0.509125 | 1.412439 | H | 6.830957 | 0.081624 | -2.191215 |
| C | 3.234245 | 1.461486 | -0.876322 | H | 7.938912 | -1.962102 | -1.330812 |
| C | 4.436551 | 1.074019 | -1.384721 | H | 6.859735 | -3.319606 | 0.438932 |

**Supplementary Data 2.2** **|** Cartesian coordinates for the structure of *Syn*-DPyB optimized by using B3LYP/6-31G(d,p)

Symbolic Z-matrix:

Charge = 0 Multiplicity = 1

| Atom | X | Y | Z | Atom | X | Y | Z |
| --- | --- | --- | --- | --- | --- | --- | --- |
| C | -0.519241 | 5.07114 | -1.097824 | C | 2.229613 | -2.03193 | -1.586312 |
| C | 0.861738 | 5.132238 | -1.279301 | C | 3.10008 | -1.689809 | -0.50795 |
| C | 1.656337 | 4.070401 | -0.854056 | C | 4.035674 | -2.655359 | -0.022716 |
| C | 1.103357 | 2.929061 | -0.249815 | C | 4.909575 | -2.288255 | 1.057172 |
| C | -0.299829 | 2.866367 | -0.063222 | C | 4.859466 | -1.047289 | 1.612838 |
| C | -1.084427 | 3.950824 | -0.493957 | C | 2.305203 | -3.316896 | -2.147851 |
| C | 2.045786 | 1.856586 | 0.200814 | C | 3.216895 | -4.252727 | -1.664619 |
| C | -0.969711 | 1.731116 | 0.641584 | C | 4.073883 | -3.927785 | -0.614814 |
| C | 2.095257 | 0.574613 | -0.400605 | H | -1.153899 | 5.893763 | -1.414138 |
| C | 3.038084 | -0.389258 | 0.08035 | H | 1.318803 | 5.998764 | -1.747984 |
| C | 3.928864 | -0.0571 | 1.14629 | H | 2.731952 | 4.108679 | -0.999486 |
| C | 3.869495 | 1.230432 | 1.697723 | H | -2.156796 | 3.913492 | -0.327753 |
| C | 2.950838 | 2.161958 | 1.227577 | H | 4.548447 | 1.496335 | 2.503539 |
| C | -0.611058 | 1.449403 | 1.967795 | H | 2.915861 | 3.151838 | 1.6736 |
| C | -1.242741 | 0.454579 | 2.70231 | H | 0.17322 | 2.03736 | 2.432303 |
| C | -2.271404 | -0.312692 | 2.137223 | H | -0.945649 | 0.269658 | 3.731044 |
| C | -2.65242 | -0.059881 | 0.784734 | H | -3.670768 | 0.568175 | -2.941697 |
| C | -1.997145 | 0.966494 | 0.029994 | H | -1.901486 | 1.910935 | -1.930816 |
| C | -3.692156 | -0.840534 | 0.191385 | H | -2.649216 | -1.519638 | 3.903588 |
| C | -4.078485 | -0.612736 | -1.163833 | H | -4.450279 | -2.852312 | 2.880886 |
| C | -3.389713 | 0.404959 | -1.90443 | H | -5.873429 | -3.379452 | 0.923676 |
| C | -2.402572 | 1.153231 | -1.339601 | H | -6.534643 | -2.966708 | -1.428536 |
| C | -2.950669 | -1.34108 | 2.874819 | H | -5.39428 | -1.212658 | -2.759014 |
| C | -3.945268 | -2.077812 | 2.309774 | H | 0.555997 | 0.935455 | -1.893772 |
| C | -4.351855 | -1.857203 | 0.949778 | H | 0.659531 | -1.291812 | -2.894306 |
| C | -5.372626 | -2.608091 | 0.344751 | H | 5.618616 | -3.026974 | 1.421453 |
| C | -5.743083 | -2.375031 | -0.978072 | H | 5.528991 | -0.780663 | 2.426417 |
| C | -5.103755 | -1.389432 | -1.726871 | H | 1.640856 | -3.574475 | -2.968271 |
| C | 1.250897 | 0.201804 | -1.503328 | H | 3.26126 | -5.24233 | -2.109917 |
| C | 1.312529 | -1.036946 | -2.063903 | H | 4.784428 | -4.661584 | -0.243795 |

**Supplementary Data 2.3** **|** Cartesian coordinates for the structure of *Anti*-DPyB (S_0_) optimized by using CAM-B3LYP-D3/6-31G**

Symbolic Z-matrix:

Charge = 0 Multiplicity = 1

| Atom | X | Y | Z | Atom | X | Y | Z |
| --- | --- | --- | --- | --- | --- | --- | --- |
| C | -2.14763 | 4.890819 | -2.34625 | C | 3.45638 | -0.13509 | -2.92446 |
| C | -0.89948 | 4.890773 | -1.73091 | C | 2.766004 | -0.92288 | -1.96739 |
| C | -0.28423 | 3.684798 | -1.41985 | C | 3.314162 | -2.16472 | -1.55075 |
| C | -0.89829 | 2.467164 | -1.71984 | C | 2.592131 | -2.95505 | -0.59061 |
| C | -2.14889 | 2.467209 | -2.35756 | C | 1.407414 | -2.53549 | -0.09152 |
| C | -2.76292 | 3.684888 | -2.65742 | C | 4.672616 | -0.5922 | -3.43807 |
| C | -0.26035 | 1.186457 | -1.3184 | C | 5.205473 | -1.80574 | -3.02111 |
| C | -2.78685 | 1.18655 | -2.75911 | C | 4.533719 | -2.58527 | -2.08765 |
| C | 0.973516 | 0.771635 | -1.84732 | H | -2.63727 | 5.828128 | -2.59069 |
| C | 1.521189 | -0.47342 | -1.43264 | H | -0.40983 | 5.828048 | -1.48639 |
| C | 0.829059 | -1.28326 | -0.49598 | H | 0.681885 | 3.672961 | -0.92511 |
| C | -0.40059 | -0.84382 | -0.00073 | H | -3.72903 | 3.673124 | -3.15218 |
| C | -0.93222 | 0.366223 | -0.41078 | H | -0.93922 | -1.46475 | 0.708924 |
| C | -2.11505 | 0.366421 | -3.66687 | H | -1.8964 | 0.690108 | -0.03249 |
| C | -2.64671 | -0.84356 | -4.07704 | H | -1.15089 | 0.690358 | -4.04519 |
| C | -3.87632 | -1.28307 | -3.58176 | H | -2.10813 | -1.46442 | -4.78679 |
| C | -4.56839 | -0.47335 | -2.64496 | H | -6.44061 | 1.696151 | 0.011981 |
| C | -4.02069 | 0.771666 | -2.23015 | H | -4.30741 | 2.474458 | -0.90739 |
| C | -5.81317 | -0.92287 | -2.11018 | H | -3.91025 | -3.13874 | -4.7067 |
| C | -6.50349 | -0.1352 | -1.15297 | H | -6.06957 | -3.90246 | -3.79829 |
| C | -5.91537 | 1.107438 | -0.73458 | H | -7.9974 | -3.53528 | -2.31195 |
| C | -4.73802 | 1.538264 | -1.24273 | H | -9.19833 | -2.1484 | -0.64862 |
| C | -4.4547 | -2.53524 | -3.98633 | H | -8.24339 | 0.014334 | 0.09376 |
| C | -5.63939 | -2.95487 | -3.48723 | H | 1.260318 | 2.474602 | -3.16983 |
| C | -6.36136 | -2.16466 | -2.52694 | H | 3.393585 | 1.696418 | -4.08917 |
| C | -7.58089 | -2.58527 | -1.99003 | H | 3.022287 | -3.90269 | -0.27963 |
| C | -8.25258 | -1.80586 | -1.05642 | H | 0.862909 | -3.13907 | 0.628741 |
| C | -7.71969 | -0.59237 | -0.63934 | H | 5.196361 | 0.014596 | -4.17106 |
| C | 1.690904 | 1.538361 | -2.8346 | H | 6.151245 | -2.14824 | -3.4289 |
| C | 2.868286 | 1.107602 | -3.34272 | H | 4.950214 | -3.53533 | -1.76581 |

**Supplementary Data 2.4** **|** Cartesian coordinates for the structure of *Syn*-DPyB (S_0_) optimized by using CAM-B3LYP-D3/6-31G**

Symbolic Z-matrix:

Charge = 0 Multiplicity = 1

| Atom | X | Y | Z | Atom | X | Y | Z |
| --- | --- | --- | --- | --- | --- | --- | --- |
| C | -1.53744 | 5.842138 | -1.18889 | C | 0.573104 | -1.38802 | -1.44701 |
| C | -0.16883 | 5.938786 | -1.41617 | C | 1.592437 | -1.06612 | -0.51449 |
| C | 0.662062 | 4.881564 | -1.06805 | C | 2.418651 | -2.09515 | 0.008726 |
| C | 0.149865 | 3.715495 | -0.49517 | C | 3.448234 | -1.74628 | 0.950743 |
| C | -1.23404 | 3.623021 | -0.25198 | C | 3.629587 | -0.46607 | 1.346149 |
| C | -2.05815 | 4.692626 | -0.60852 | C | 0.392465 | -2.7195 | -1.82963 |
| C | 1.08407 | 2.62346 | -0.11615 | C | 1.201796 | -3.72177 | -1.30972 |
| C | -1.81892 | 2.428667 | 0.412017 | C | 2.206799 | -3.41388 | -0.4007 |
| C | 0.93579 | 1.309904 | -0.59935 | H | -2.1972 | 6.6621 | -1.45411 |
| C | 1.778363 | 0.285437 | -0.09399 | H | 0.25067 | 6.832566 | -1.86694 |
| C | 2.797651 | 0.594142 | 0.844319 | H | 1.730225 | 4.942159 | -1.25189 |
| C | 2.95793 | 1.918008 | 1.2557 | H | -3.12252 | 4.614092 | -0.41091 |
| C | 2.108871 | 2.908674 | 0.786364 | H | 3.741779 | 2.160791 | 1.967245 |
| C | -1.35505 | 2.070203 | 1.678375 | H | 2.213719 | 3.927842 | 1.145549 |
| C | -1.83062 | 0.947506 | 2.332114 | H | -0.59172 | 2.683792 | 2.144159 |
| C | -2.7861 | 0.126589 | 1.730203 | H | -1.45594 | 0.689401 | 3.318048 |
| C | -3.26466 | 0.464274 | 0.438589 | H | -4.50187 | 1.302352 | -3.17092 |
| C | -2.78599 | 1.628214 | -0.22216 | H | -2.88716 | 2.769989 | -2.07251 |
| C | -4.21216 | -0.38915 | -0.20339 | H | -2.91109 | -1.29099 | 3.368627 |
| C | -4.67313 | -0.08707 | -1.51049 | H | -4.56947 | -2.74453 | 2.269584 |
| C | -4.1581 | 1.084441 | -2.16379 | H | -5.97807 | -3.26079 | 0.320733 |
| C | -3.26704 | 1.898101 | -1.55397 | H | -6.78526 | -2.71526 | -1.9571 |
| C | -3.2877 | -1.05436 | 2.377796 | H | -5.94588 | -0.69703 | -3.12562 |
| C | -4.19693 | -1.85264 | 1.774454 | H | -0.65536 | 1.743887 | -2.01373 |
| C | -4.68955 | -1.55152 | 0.457839 | H | -0.9996 | -0.56821 | -2.706 |
| C | -5.61592 | -2.37327 | -0.19003 | H | 4.078092 | -2.5416 | 1.33872 |
| C | -6.06586 | -2.06627 | -1.46793 | H | 4.408803 | -0.21005 | 2.058273 |
| C | -5.59749 | -0.93488 | -2.12474 | H | -0.39228 | -2.96 | -2.54086 |
| C | -0.04817 | 0.956705 | -1.58734 | H | 1.048734 | -4.75177 | -1.61586 |
| C | -0.2314 | -0.32347 | -1.97842 | H | 2.839465 | -4.19855 | 0.003945 |

**Supplementary Data 2.5** **|** Cartesian coordinates for the structure of *Anti*-DPyB (S_1_) optimized by using CAM-B3LYP-D3/6-31G**

Symbolic Z-matrix:

Charge = 0 Multiplicity = 1

| Atom | X | Y | Z | Atom | X | Y | Z |
| --- | --- | --- | --- | --- | --- | --- | --- |
| C | -2.24185 | 4.820401 | -2.1574 | C | 3.570332 | -0.17733 | -3.00498 |
| C | -0.85696 | 4.85011 | -1.91121 | C | 2.940583 | -0.8826 | -1.94292 |
| C | -0.15807 | 3.673139 | -1.74746 | C | 3.529107 | -2.0814 | -1.44843 |
| C | -0.8158 | 2.429813 | -1.80253 | C | 2.862055 | -2.79761 | -0.41083 |
| C | -2.20436 | 2.396046 | -2.14185 | C | 1.667676 | -2.36287 | 0.086265 |
| C | -2.90172 | 3.61534 | -2.26232 | C | 4.774939 | -0.67118 | -3.54087 |
| C | -0.15773 | 1.195658 | -1.39852 | C | 5.343369 | -1.83257 | -3.04438 |
| C | -2.81746 | 1.126637 | -2.50464 | C | 4.732013 | -2.53328 | -2.01078 |
| C | 1.112987 | 0.776053 | -1.90867 | H | -2.78786 | 5.748079 | -2.29448 |
| C | 1.708829 | -0.41503 | -1.40416 | H | -0.34288 | 5.801536 | -1.82398 |
| C | 1.053519 | -1.17958 | -0.39825 | H | 0.897508 | 3.695471 | -1.49856 |
| C | -0.21602 | -0.75545 | 0.062752 | H | -3.95556 | 3.593016 | -2.51952 |
| C | -0.80357 | 0.373245 | -0.44027 | H | -0.72272 | -1.34065 | 0.823778 |
| C | -2.12045 | 0.277321 | -3.40551 | H | -1.77599 | 0.679606 | -0.0717 |
| C | -2.67429 | -0.86804 | -3.90691 | H | -1.13906 | 0.582706 | -3.747 |
| C | -3.97399 | -1.27645 | -3.52158 | H | -2.12719 | -1.46753 | -4.62768 |
| C | -4.68973 | -0.48192 | -2.58172 | H | -6.60296 | 1.556861 | 0.135102 |
| C | -4.11805 | 0.71447 | -2.05925 | H | -4.38504 | 2.277513 | -0.5873 |
| C | -5.98066 | -0.9048 | -2.15664 | H | -4.01559 | -3.04077 | -4.76302 |
| C | -6.69753 | -0.15309 | -1.18562 | H | -6.2703 | -3.74382 | -4.06388 |
| C | -6.07774 | 1.001733 | -0.63622 | H | -8.28278 | -3.37958 | -2.68569 |
| C | -4.83872 | 1.409525 | -1.05032 | H | -9.52387 | -2.05229 | -1.00854 |
| C | -4.57424 | -2.45096 | -4.04212 | H | -8.52056 | -0.00797 | -0.043 |
| C | -5.82305 | -2.84317 | -3.6534 | H | 1.267335 | 2.298885 | -3.43821 |
| C | -6.56254 | -2.0885 | -2.69642 | H | 3.387133 | 1.503916 | -4.35521 |
| C | -7.84055 | -2.47914 | -2.26929 | H | 3.314641 | -3.71054 | -0.03525 |
| C | -8.53634 | -1.7315 | -1.3254 | H | 1.156283 | -2.93025 | 0.858375 |
| C | -7.97754 | -0.58575 | -0.78475 | H | 5.247985 | -0.13289 | -4.35674 |
| C | 1.750143 | 1.435325 | -2.99599 | H | 6.270209 | -2.20396 | -3.47006 |
| C | 2.931089 | 0.985621 | -3.51714 | H | 5.178244 | -3.44864 | -1.63366 |

**Supplementary Data 2.6** **|** Cartesian coordinates for the structure of *Syn*-DPyB (S_1_) optimized by using CAM-B3LYP-D3/6-31G**

Symbolic Z-matrix:

Charge = 0 Multiplicity = 1

| Atom | X | Y | Z | Atom | X | Y | Z |
| --- | --- | --- | --- | --- | --- | --- | --- |
| C | -1.28689 | 6.244546 | -1.21922 | C | -0.2107 | -1.03535 | -2.42823 |
| C | -0.07347 | 5.951078 | -1.83166 | C | 0.339547 | -1.09768 | -1.11526 |
| C | 0.57777 | 4.750518 | -1.55743 | C | 0.472807 | -2.35564 | -0.47032 |
| C | 0.011832 | 3.841119 | -0.67042 | C | 1.003209 | -2.39482 | 0.844449 |
| C | -1.23078 | 4.124826 | -0.0757 | C | 1.376371 | -1.24759 | 1.490403 |
| C | -1.86756 | 5.332799 | -0.34121 | C | -0.62648 | -2.23335 | -3.0543 |
| C | 0.630412 | 2.545103 | -0.26049 | C | -0.46412 | -3.46008 | -2.42423 |
| C | -1.77479 | 3.041203 | 0.795008 | C | 0.056117 | -3.52835 | -1.14424 |
| C | 0.457541 | 1.36687 | -1.04364 | H | -1.79016 | 7.181811 | -1.43454 |
| C | 0.701626 | 0.098954 | -0.43342 | H | 0.371402 | 6.661144 | -2.52151 |
| C | 1.225895 | 0.024327 | 0.882207 | H | 1.531941 | 4.521177 | -2.02209 |
| C | 1.544076 | 1.227032 | 1.560022 | H | -2.82518 | 5.547162 | 0.123314 |
| C | 1.236481 | 2.444314 | 0.996358 | H | 1.985289 | 1.174705 | 2.54986 |
| C | -1.57522 | 3.061147 | 2.179062 | H | 1.391771 | 3.355028 | 1.565199 |
| C | -1.70956 | 1.917037 | 2.936343 | H | -1.19835 | 3.970267 | 2.636757 |
| C | -2.06914 | 0.686847 | 2.331732 | H | -1.48671 | 1.934265 | 3.998447 |
| C | -2.42762 | 0.681984 | 0.960339 | H | -3.48131 | 0.67228 | -2.79392 |
| C | -2.34851 | 1.880874 | 0.193673 | H | -2.75656 | 2.768009 | -1.73015 |
| C | -2.78502 | -0.53904 | 0.326653 | H | -1.76524 | -0.53578 | 4.086668 |
| C | -3.15361 | -0.54987 | -1.05075 | H | -2.29262 | -2.66083 | 2.963068 |
| C | -3.17389 | 0.673424 | -1.75268 | H | -2.95826 | -3.90484 | 0.930151 |
| C | -2.76747 | 1.849057 | -1.15572 | H | -3.60402 | -3.91096 | -1.45289 |
| C | -2.04734 | -0.54283 | 3.038162 | H | -3.73411 | -1.79248 | -2.72068 |
| C | -2.3431 | -1.72506 | 2.414121 | H | -0.21544 | 2.34906 | -2.84217 |
| C | -2.72065 | -1.76022 | 1.046769 | H | -0.76013 | 0.278504 | -4.04676 |
| C | -3.01351 | -2.97222 | 0.376782 | H | 1.105419 | -3.3583 | 1.335175 |
| C | -3.38652 | -2.97089 | -0.95571 | H | 1.777416 | -1.29601 | 2.498362 |
| C | -3.44406 | -1.78569 | -1.67493 | H | -1.04077 | -2.18429 | -4.05685 |
| C | -0.04823 | 1.391098 | -2.36386 | H | -0.77698 | -4.36896 | -2.92887 |
| C | -0.36187 | 0.229441 | -3.03752 | H | 0.171522 | -4.48716 | -0.64797 |
